# Glioblastoma Treatments: An Account of Recent Industrial Developments

**DOI:** 10.3389/fphar.2018.00879

**Published:** 2018-09-13

**Authors:** Edouard Alphandéry

**Affiliations:** ^1^Institut de Minéralogie, de Physique des Matériaux et de Cosmochimie, UMR 7590 CNRS, Sorbonne Universités, UPMC, University Paris 06, Paris, France; ^2^Nanobacterie SARL, Paris, France

**Keywords:** brain cancer, industry, drug development, market, preclinical model

## Abstract

The different drugs and medical devices, which are commercialized or under industrial development for glioblastoma treatment, are reviewed. Their different modes of action are analyzed with a distinction being made between the effects of radiation, the targeting of specific parts of glioma cells, and immunotherapy. Most of them are still at a too early stage of development to firmly conclude about their efficacy. Optune, which triggers antitumor activity by blocking the mitosis of glioma cells under the application of an alternating electric field, seems to be the only recently developed therapy with some efficacy reported on a large number of GBM patients. The need for early GBM diagnosis is emphasized since it could enable the treatment of GBM tumors of small sizes, possibly easier to eradicate than larger tumors. Ways to improve clinical protocols by strengthening preclinical studies using of a broader range of different animal and tumor models are also underlined. Issues related with efficient drug delivery and crossing of blood brain barrier are discussed. Finally societal and economic aspects are described with a presentation of the orphan drug status that can accelerate the development of GBM therapies, patents protecting various GBM treatments, the different actors tackling GBM disease, the cost of GBM treatments, GBM market figures, and a financial analysis of the different companies involved in the development of GBM therapies.

## Introduction

Glioblastoma multiform (GBM) is a malignant tumor originating from glial cells. It is the most frequent brain tumor, representing 30% of all central nervous system tumors (CNST), 45% of malignant CNST and 80% of primary malignant CNST. It leads to 225,000 deaths per year in the entire word. It has an incidence of 5 per 100,000 persons, affects 1.5 times more men than women, and is diagnosed at an average age of 64 (Bush et al., 2017). Due to the relatively limited number of people suffering from GBM, it is difficult to determine with certainty the causes of this disease. The only well-established GBM risk factor is exposure to radiation. Radiofrequency electromagnetic fields such as those produced by mobile phones have been classified as IIB and may also play a role in GBM appearance (Armstrong et al., 2011). By contrast to other types of cancers, it appears uncertain that GBM incidence can be decreased by changing certain environmental factors such as alcohol or tobacco consumption. Since the majority of GBM appear for the first time, i.e., only ~40% originates from tumors of lower grades, it also seems rather uneasy to anticipate GBM from the presence of another disease or condition.

Among the four different forms of glioma, grade IV corresponds to GBM. It is the most deadly grade, due to its frequent relapse and resistance to all current therapies and is the topic of this review. GBM current standard of care (SOC) includes maximal safe resection followed by radiotherapy and chemotherapy using temozolomide (TMZ). Such treatment hardly increases patient survival and leads to a median overall survival (OS) of only 12–18 months following diagnosis (Stupp et al., 2005; Wen and Kesari, 2008).

Efficient treatment against GBM is difficult to develop for a series of reasons that are summarized below. First, GBM is characterized by many dysregulated pathways that can hardly be all blocked and repaired at the same time with a single therapy (Alifieris and Trafalis, 2015). Second, GBM partly consists of infiltrating cells that cannot easily be all removed by surgery. Full tumor resection would require very precise imaging and surgical tools to enable the visualization and removal of all GBM infiltrating cells. Third, GBM early diagnosis, which may improve treatment efficacy by enabling the removal of tumors of small sizes, is not carried out routinely. In fact, the first signs of GBM, such as vomiting and strong headache, often appear at a late stage of this disease, and sensitive imaging techniques, such as MRI, which could possibly enable early diagnosis, still seem too expensive to be carried out on a regular basis over the whole population. Fourth, the optimization of a clinical protocol for GBM treatment requires the use of an accurate and representative preclinical GBM model. Different types of mouse and rats models have been developed, each one with its own advantages and drawbacks. It therefore appears necessary to test GBM drug efficacy on a combination of several of these models to grasp sufficient information for optimal design of the clinical protocol. Furthermore, mouse and rats GBM tumors are typically ~10^3^-10^4^ smaller than human GBM. The optimization of the clinical protocol would therefore certainly benefit from preclinical efficacy tests carried out on larger animals such as dogs. Fifth, the blood brain barrier (BBB) often prevents drugs from efficiently reaching glioblastoma cells, and methods to enable drugs to efficiently cross the BBB should therefore be developed.

Here, I review the different drugs and medical devices, which are under development or commercialized by companies, have been pre-clinically or clinically tested, most frequently involve medical teams, and either result in direct GBM cell destruction or are part of a GBM treatment protocol, e.g., through GBM imaging. I focus on GBM treatments that have been the subject of at least one publication listed in the pubmed search database. I also discuss several scientific, societal, and industrial issues related to early GBM diagnosis, an adapted preclinical model, different methods to yield efficient drug delivery to GBM tumor, program to accelerate the development of GBM therapies, patents protecting various GBM treatments, the different actors tackling GBM, the cost associated with GBM treatment, GBM market figures, and finally a financial analysis of the different companies involved in the development of GBM treatment.

## The different GBM treatments commercialized or under development

**Figure 1:**
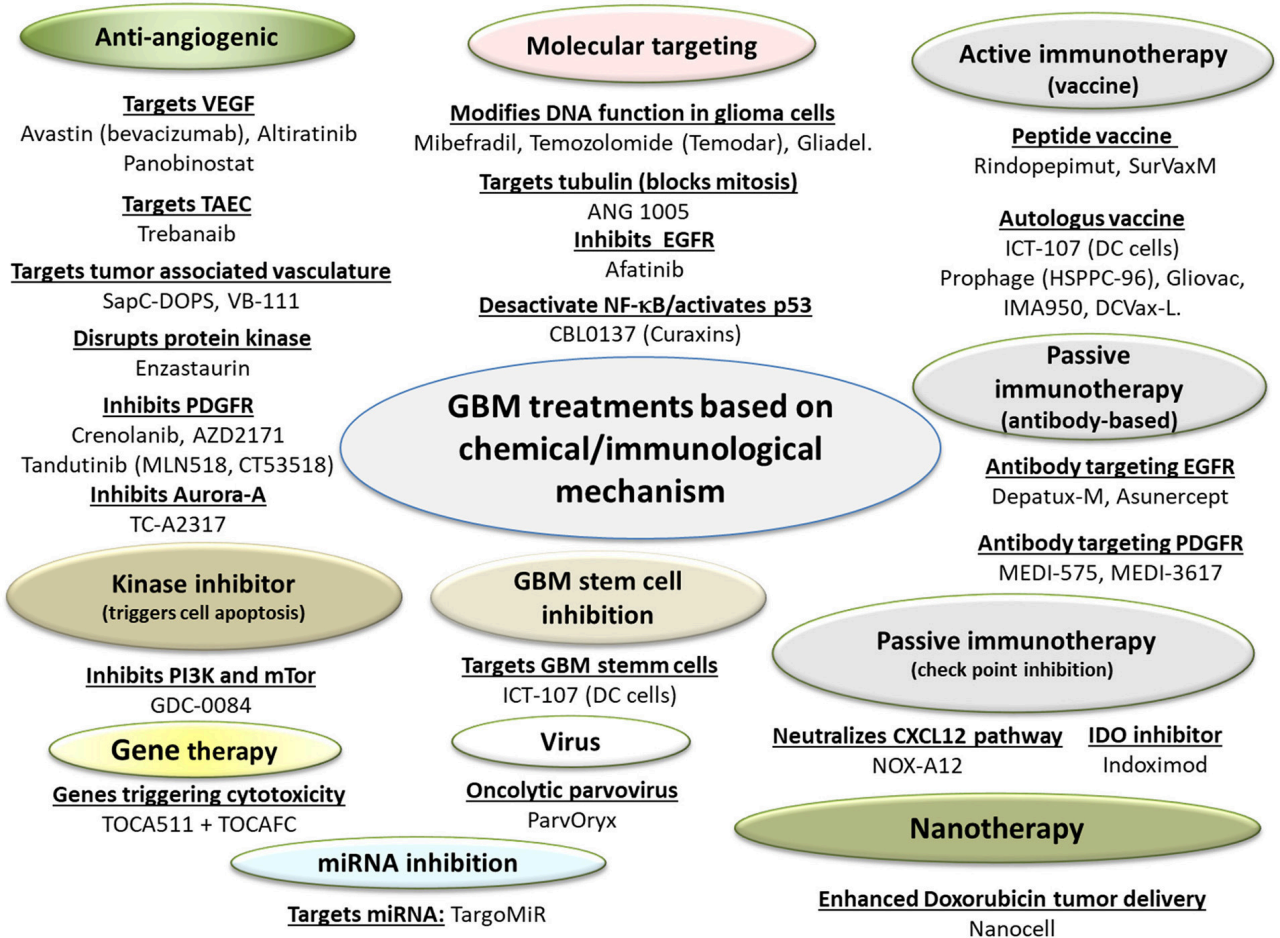
A schematic diagram presenting the different GBM treatments relying on chemical and immunological mechanisms. These treatments are classified as drugs, since their dominant mode of action is immunological, metabolic, or pharmacological. GBM drugs with their associated mode of action are listed.

**Figure 2:**
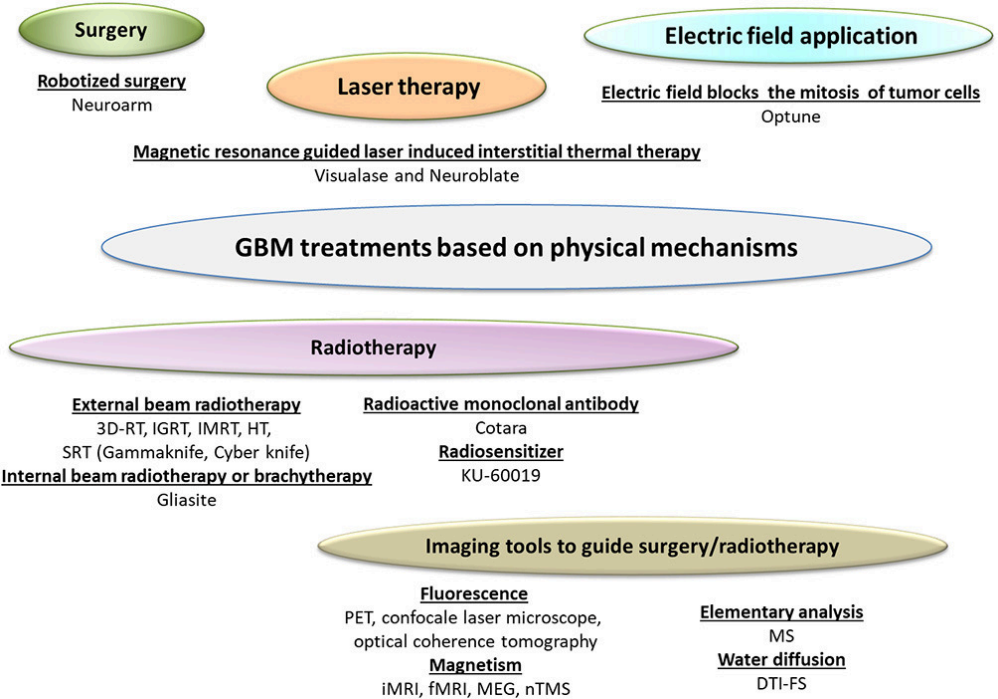
A schematic diagram presenting the different GBM treatments relying on physical mechanisms. These treatments (except Cotara and KU-60019) are classified as medical devices, since their dominant mode of action is not immunological, metabolic, or pharmacological. These GBM treatments with their associated mode of action are listed.

The different drugs and medical devices used for GBM Treatments are summarized in Figures 1 and 2. The type of drug, name of company developping it, proposed drug made of section are indicated in Table 1. The preclinical/clinical result obtained with these drug are listed in Table 2.

**Table 1:** A list of the different GBM therapies, associated drug names, companies in charge of their development or commercialization, as well as drug proposed mode of action.

**Therapy**	**Drug type**	**Company name**	**Proposed modes of action**
Afatinib	ErbB family inhibitor	Boehringer Ingelheim	Binds to ErbB receptor and inhibits EGFR activity (glioma cell proliferation).
AFM 21	Bivalently binding TandAb	Affimed therapeutics	TandAbs recruit either cytotoxic T- or NK-cells that eliminate cancer cells with EGFRvIII+.
Aldoxorubicin	Cytotoxic	CytRx	Doxorubicin combined with a linker that binds to circulating albumin. Tumors concentrate albumin, thus increasing the delivery of the linker molecule with the attached doxorubicin to tumor sites. Doxorbucin selectively released at tumor site due to its acidic environment.
Altiratinib	Inhibitor of MET/TIE2/VEGFR2	Deciphera	Prevent or delay bevacizumab-mediated resistance mechanisms.
ANG1005	Paclitaxel-peptide drug conjugate	Angiochem	Paclitaxel modified to cross BBB (Bertrand et al., 2011). It targets tubulin and blocks mitosis.
APG101 (Asunercept)	Antibody conjugated with CD95	Apogenix	Binds and neutralizes CD95L responsible in high motility of glioma cells Merz et al., 2015.
AV0113	Immunotherapy	Activartis Biotech GMBH	Dendritic cell (DC)-based vaccine composed of autologous monocyte-derived DCs exposed to LPS express IL-12 and activate NK cells T-cells against tumor cells.
Avastin (bevacizumab)	Antiangiogenic	Roche	Neutralizing antibody targeting vascular endothelial growth factor (VEGF).
BiCNU	Carmustine	Emcure Pharma Uk Ltd	Dialkylating agent forms interstrand crosslinks in DNA, which prevents DNA replication and DNA transcription.
CBL0137 (curaxins)	Similar to anti-malarial agent	Incuron	Different structure from the tested anti-malarials but similar activation of p53 (tumor suppressor) and suppressing NF-κB (pro-survival transcription factor) without inducing genotoxicity.
Crenolanib	Inhibitor of PDGFRα/β	Arog Pharamceuticals	Inhibtits PDGFR (a type I kinase) that drive glioblastoma growth.
DCVax-L	Vaccine (autologous tumor antigen and patient DC)	Northwest biotherapeutics	Tumor antigens and DC, obtained by surgical resection and leukapheresis, respectively, DCs are mixed and injected back to the patient, allowing DCs to present their surface tumor antigens to the CD4 and CD8 T-cells of the immune system, leading to the activation of T-cells against the tumor.
Depatux-M; ABT-414	EGFR-targeted antibody-drug conjugate	Abbvie	preferentially binds glioma cells with EGFR amplification, is internalized and releases a potent antimicrotubule agent, monomethyl auristatin F (MMAF).
Enzastaurin	Anti-angiogenic	Elly Lilly	Disrupts the protein kinase C (PKC), which is essential for angiogenesis and tumor growth.
Gama Knife	Stereotactic radio-surgery	Elekta	Cobalt-60 machine generating gamma rays over a precise delineated region containing the tumor (tumor size < 3 cm).
GDC-0084	Inhibitor of PI3K kinase	Novogen	An inhibitor of phosphoinositide-3-kinase (PI3K) and mTOR, which is able to cross the BBB
Gliadel	Carmustine wafer	Eisai	Wafer containing carmustine implanted into the brain following surgical removal of malignant glioma allows direct delivery of Carmustine to the tumor site.
Gliovac ERC1671	Immunotherapy	ERC	Autologous and allogeneic tumor cells generated from the glioma tumor tissues of three different donor cancer patients, and the lysates of all of these cells. This mixture is injected to stimulate the patient's immune system against the tumor cells.
GMCI (Gene-mediated cytotoxic immunotherapy)	Vaccine-like	Advantagene	Activates adaptive and innate immunity.
ICT-107	Autologous vaccine	Immunocellular	Targets tumor antigens highly expressed on glioblastoma cancer stem cells.
IMA950	Vaccine	Immatics Biotechnologies	11 different HLA-restricted tumor-associated peptides over-expressed on the surface of glioblastoma tumors trigger the immune system to recognize and kill tumor cells while leaving healthy cells unharmed.
Indoximod	IDO inhibitor	Newlinkgenetics	Inhibits IDO (indoleamine 2,3-dioxygenase) that inactivates NK (natural killer) cells and generates Tregs (regulatory T cells).
KML001	A telomere targeting drug	Komipharm International	Sensitizes glioblastoma cells to temozolomide chemotherapy and radiotherapy through DNA damage and apoptosis.
MEDI-575	human IgG2 Antibody	MedImmune	High affinity and specificity for human PDGFRα, reducing the growth of GBM tumors.
Mibefradil	Cytotoxic	Cavion	Inhibits Cav3 (T-type calcium channel essential for external calcium entry in glioma cells), hampers a glioma cell ability to repair double-strand DNA breaks and causes cancer cell cycle arrest and apoptosis.
Nanocell	cytotoxic	EnGeneIC	Nanocellulars (minicell) contain Doxorubicin and target EGFR overexpressed in tumors via minicell-surface attached bispecific proteins (Vectibix).
Neuroblate	MRgLITT	Monteris	Both diffusing (FullFire) and side-firing (SideFire) directional laser delivery probes (LDPs). Pulsed laser of 1,064 nm with maximum power of 12 W also including a controlled cooling mechanism. Temperature monitored by real-time MR thermography (Lagman et al., 2017).
NOX-A12	Neutralizes CXCL12	Noxxon	Neutralizes chemokine CXCL12 pathway, which promotes cancer cell survival, facilitates tumor recurrence and metastasis, and promoting angiogenesis. NOX-A12 also fights tumors by: (i) breaking tumor protection against immune cells T-cells, (ii) blocking tumor repair, (iii) exposing hidden tumor cells.
Optune	Tumor-treating fields	Novocure	Generates an alternating current (100–300 kHz) that alters tumor cell polarity and blocks tumor cell mitosis.
ParvOryx	Virus	Oryx	Oncolytic parvovirus H1 (H-1PV) that infects and lyses GBM tumor cells. Due to its small size, it crosses the BBB. It does not affect normal cells and is not pathogenic to humans. I allows for both intratumor and intravenous administration as well as repeated application.
Prophage (G100 and G200)	Vaccine (patient specific)	Agenusbio	Use of the heat shock protein gp96 (HSPPC-96), purified from tumor tissue inducing immune response against the tumor.
PSMA ADC	Antiangiogenic	Ambrx	PSMA-targeted monoclonal antibody conjugated with microtubule disrupting agent monomethyl auristatin E (MMAE) targets PSMA, transmembrane peptidase upregulated on endothelial GBM cells.
Rindopepimut (CDX-110)	Peptide vaccine	Celldex	Anti-EGFRvIII immune responses. EGFRvIII (most common in primary glioblastoma tumors) is a tumor-specific epitope expressed on ~20–30% of GBMs, containing a tyrosine kinase that has pro-oncogenic effects.
SapC-DOPS	Anti-angiogenic.	Bexion pharmaceuticals	Affinity for phosphatidylserine in the outer membrane of tumor-associated vasculature of GBM.
Selinexor (KPT-330)	Small molecule	Karyopharm Therapeutics	Selinexor inhibits nuclear export protein XPO1 that inactivates tumor suppressor protein.
TC-A2317	Aurora-A inhibitor	Takeda	Inhibits Aurora-A, a serine/threonine kinase that drives GBM cell cycle progression.
Temodar (Temozolomide)	alkylating agent	Merck	Breaks DNA double-strand, causing cell cycle arrest and cell death.
Toca 511 + Toca FC	Gene therapy + prodrug	Tocagen	Toca 511 encodes and delivers cytosine deaminase (CD) gene to tumor. Toca FC induces transformation of 5-fluorocytosine in 5-fluorouracil in tumor cells having expressed CD gene.
Trebanaib AMG 386	Antiangiogenic	Amgen	Peptide-Fc fusion protein that blocks angiopoietin-Tie2 signaling and inhibits proliferation of Tumor Associated Endothelial Cells.
VAL-083	chemotherapy	DelMar Pharmaceuticals	Cytotoxicity (claimed to be larger than for TMZ), can overcome resistance associated with MGMT (O6-DNA methylguanine methyl-transferase), a DNA repair enzyme that causes resistance to TMZ.
VB-111	Immunotherapy	VBLRX	Combination of tumor vasculature blockade with anti-tumor immune response.
Visualse	MRgLITT	Medtronic	Laser at 980 nm with maximum power of 15 W used to heat and destroy tissue during neurosurgery. Probe tip cooled down by saline circulation. Temperature monitored by real-time MR thermography, (Lagman et al., 2017).

**Table 2:** A list of the different GBM drugs, with a summary of the publically available preclinical and/or clinical results.

**Drug name**	**Preclinical/Clinical/results**
Afatinib	Manageable safety profile but limited activity (Reardon et al., 2014b).
AFM21	N.A.
Aldoxorubicin	Tumor growth delay observed in mice bearing U87-Luc tumors after injection of Aldoxorubicin (Marrero et al., 2014).
Altiratinib	Tumor growth delay observed in mice bearing GSC11 and GSC17 glioblastoma (Piao et al., 2016).
ANG1005	Mice bearing U87 MG glioblastoma treated with ANG1005 display enhanced tumor growth delay compared with those treated with free paclitaxel (regina2008).
APG101 (Asunercept)	Phase II on 9 patients with recurrent glioblastoma: PFS at 6 months were 20.7% for RT + APG101 compared with 3.8% for RT alone. Improved survival warrants further studies for confiration (Wick et al., 2014).
AV0113	N.A.
Avastin (bevacizumab)	Partial antitumor activity in mice with sarcoma tumors (Presta et al., 1997). Phases II and III: No improvement in survival when Avastin is used as first and second-line therapy, and both in association with cytotoxic treatment or alone (Lombardi et al., 2017).
BiCNU	N.A.
CBL0137	IV injection of CBL0137 ± TMZ in mice bearing U87MG/A107 GBM Increases mouse maximum survival by 10–60 days.
Crenolanib	Glioma cell inhibition.
DCVax-L	Phase II suggests efficacy with 33% of patients reaching or exceeding median survival of 48 months and 27% reaching or exceeding median survival of 72 months. Two patients reached a survival of more than 10 years (Polyzoidis and Ashkan, 2014). Phase III on 331 patients on going.
Depatux-M; ABT-414	Clinical trial on 66 patients leads to PFS at 6 months of 28.8%.
Enzastaurin	Phase III: 266 patients with recurrent glioblastoma treated. Enzastaurin well tolerated and better hematologic toxicity profile than lomustine but no superior efficacy compared with lomustine (Wick et al., 2010).
ERC-1671	One patient receiving ERC-1671 survived for 10 months after the vaccine administration without any other adjuvant therapy and died of complications due his previous therapies (Bota et al., 2015). For 9 patients treated with ERC-1671, 6-month (26 weeks) survival for the nine Gliovac patients was 100 vs. 33% in control group (Schijns et al., 2015).
Gama Knife	Clinical results are too preliminary. Survival benefit still needs to be demonstrated in a phase III clinical study (Elaimy et al., 2013).
GDC-0084	Mice bearing U87 MG glioblastoma injected with GDC-0084 exhibited tumor growth delay (Heffron et al., 2016).
Gliadel	3 clinical trials with increased survival by 6–13 months. 3 clinical trials without increased survival, (Zhang et al., 2013). MA: 1998.
GMCI	80% of mice bearing GL-261 tumors treated with PD-1 and GMCI cured (Speranza et al., 2018).
ICT-107	Phase I: prolonged overall survival and PFS (preliminary data, Phupahnich et al., 2013).
IMA950	Clinical Trial: 49 patients with GBM treated with IMA950. PFS was 74% at 6 months and 31% at 9 months.
Indoximod	Tumor growth delay observed in mice bearing GL-261 glioblastoma tumors injected with Indoximod (Hanihara et al., 2016).
KML001	Clonogenic survival of GBM cells was significantly decreased by the combination of KML001 and TMZ or irradiation (Woo et al., 2014).
MEDI-575	Phase II on 56 patients receiving MEDI-575 showed that MEDI-575 was well tolerated but had limited clinical activity (Phupahnich et al., 2013).
MgLITT (Neuroblate Visualase)	Treatment relatively well tolerated. Minimal BBB permeation (Carpentier et al., 2012). In 16 patients with GBM, Improved survival by 2 months (survival benefit warrants further study) (Schwarzmaier et al., 2006).
Mibefradil	Well tolerated and activity on some patients (Holdhoff et al., 2017).
Nanocell	First in man shows that nanocell was well tolerated in patients bearing glioblastoma (Whittle et al., 2015).
NOX-A12	Mice bearing G12 GBM tumors injected with B-20 and NOX-12 led to an increase in maximum survival by 15 days.
Optune	Increase in time to disease progression from 13 to 26 weeks and of PFS6 from 15 to 50% and OS from 6 to 14.7 months (Saria and Kesari, 2016).
Panobinostat	Phase II on 15 patients, Panobinostat well tolerated, but no significant improvement in PFS6 compared with SOC (Lee et al., 2015).
Parvovirus	In a phase I study, parvovirus was well tolerated and immune response was observed (Geletneky et al., 2017).
Prophage	Phase II: Prophage + radiation and temozolomide lead to: (i) a 146% increase of PFS (17 months compared with 6.9 months for SOC), (ii) a 60% increase of OS (23.3 months compared with 14.6 months for SOC), (Chakraborty et al., 2016).
PSMA ADC	Phase II on 6 patients (trial NCT01856933), efficacy not observed (Elinzano et al., 2016).
Rindopepimut (CDX-110)	Phase II: demonstrating significantly increase by 10 months in PFS, minimal adverse effects (Babu and Adamson, 2012). Phase III (trial NCT01480479) did not confirm increases in PFS observed during phase II (Desaia et al., 2016; Gerstner, 2017; Weller et al., 2017).
SapC-DOPS	Tumor growth delay in mice bearing U87 (Wojton et al., 2013; Blanco et al., 2014, 2015).
Selenexor	Mice bearing patient derived GBM genograft model inhibit tumor growth delay following Selenexor injection (Green et al., 2014).
SurVaxM	Among 9 patients treated, 7 survived more than 12 months. Requires more clinical data to conclude about treatment efficacy (Fenstermaker et al., 2016).
Tandutinib	Phase II was closed due to a lack of efficacy (Batchelor et al., 2016).
TC-A2317	GB neurosphere cells treated with alisertib for short periods undergo apoptosis (Van Brocklyn et al., 2014).
Temodar (Temozolomide)	Radiotherapy + Temozolomide: 2 months increase in overall survival, 15% increase in the percentage of patients alive after 2 years (Lee, 2017). Efficacy of TMZ limited due to MGMT that repairs DNA in tumor cells and reduces the effect of this alkylating agent and overexpression of EGFR. MA:2009.
Toca 511 + FC	High percentage of mice (40–100% depending on injected dose) bearing U87, Tu-2449 glioblastoma are alive 3–10 months following tumor cell implantation (Hiraoka et al., 2017).
Trebanaib AMG 386	Phase II on 48 patients, treatment well tolerated but no improvement in survival (Reardon et al., 2018).
VAL-083	Clinical trial (NCT02717962) ongoing.
VB-111	Tumor growth delay in mice bearing U87-MG injected with VB-111 (Gruslova et al., 2015).

*M.A, Market Authorization; NA, Not Available*.

### Surgery

Surgery is feasible in ~60% of all GBM patients (Stark et al., 2012). For these patients, it represents the initial treatment and usually consists in maximal safe surgical resection. It leads to the best treatment outcome when the extent of tumor resection is the largest (Stuschke and Thames, 1997; Hess, 1999; Stummer et al., 2008). GBM cells have a tendency to infiltrate normal parenchyma, spread into the ventricles, and to remain in the post-surgical cavity where they can form a new tumor within 2–3 cm of the tumor margin. One major difficulty resides in removing glioma cells remaining within the tumor margin without producing adverse effects such as unintentional damage to surrounding healthy tissues, possibly leading to language and motor deficits. Surgical methods and associated imaging techniques, which are under development to improve the efficacy of surgery and reduce its side effects, are described below.

#### Methods used for maintaining patients awake during surgery

**Awake craniatomy** (AC) is a method that maintains patient awake during the surgical operation that can be carried out using a neuronavigation system, such as the Stealth Station developed by Medtronic (Parney et al., 2010). Compared with general anesthesia, AC leads to better GBM tumor resection and postoperative functional status, and to reduction in morbidity (Eseonu et al., 2017). Furthermore, AC enables to decrease hospitalization time by 3 days, hence reducing the cost of a surgical operation (Eseonu et al., 2017). However, AC remains relatively complex to achieve, requiring the presence of a multidisciplinary team composed of surgeons, anesthesiologists, and neurologists.

#### Surgical robot

**Neuroarm**, commercialized by Integrated Surgical Systems, is a magnetic resonance imaging (MRI) compatible microsurgery and stereotaxic system that enables the surgeon to see GBM lesions and remove them almost simultaneously. Furthermore, due to its surgical tools that are automatically controlled by measuring the forces that they apply on tumor tissue, Neuroarm can precisely remove part of the GBM tumor (Maddahi et al., 2016). This robot can be useful to reduce surgeon tasks and fatigue (Sutherland et al., 2015), but its use was not yet shown to improve patient survival compared with conventional surgery (Maddahi et al., 2016).

#### Imaging techniques used during brain surgery

Standard imaging techniques such as MRI and computed tomography (CT) can be used to obtain brain maps before a surgical operation. However, since they are not established during the surgical operation, they don't precisely represent brain status or structure during tumor resection, leading to the so-called brain shifts, i.e., discrepancies that have led to numerous side effects such as deformations of cortical and subcortical structures, loss of cerebrospinal fluid (CSF), or brain edema. To improve brain tumor imaging, the following real time techniques have therefore been developed.

##### Fluorescent imaging systems

**Positron emission tomography (PET)**, using for example Siemens EXACT/HR or ADVANCE NXi positron emission tomograph commercialized by Siemens and GE respectively, is a molecular imaging technique that provides information about molecular processes taking place in GBM tumor. In PET, the nucleus of radioisotopes emits positrons that annihilate when they meet electrons, producing photons that are counted on a detector unit. Different types of radioisotopes are used to monitor specific molecular transformations taking place in GBM, for example Fluoro-2-deoxy-D-glucose ([18F]FDG) for measuring glucose metabolism, radiolabeled amino acids ([11C]methionine), and aromatic amino acid ([18F]fluorotyrosine, [18F]fluoromethyltyrosine, [18F]fluorodopa) to monitor amino acid transport as well as protein synthesis that are enhanced in glioblastoma tumor, Nitroimidazole derivatives ([18F]fluoromisonidazole and [18F]FAZA38) to detect tumor hypoxia, choline analog ([18F]fluorocholine) produced by high grade glioma and their metastases. With the help of this large variety of radiotracers, PET is able to identify malignant regions with a relatively high resolution (1.5 mm at best), and can therefore guide the surgeon during glioma resection (Chiang et al., 2018).

**Confocal Laser Endo-microscope (CLEM)**, for example the Cellvizio system developed by Mauna Kea Technologies, is a fluorescent detection method, which was used during GBM surgery in combination with different contrast agents (5-aminolevulinic acid and fluorescein) and enables to distinguish between healthy and glioma cells (Pavlov et al., 2016).

**Optical Coherence Tomography (OCT)**, which can be carried out with a Sirius 713 Tomograph developed by 4Optics AG, uses near-infrared light penetrating at a depth of up to several millimeters that is reflected to generate cross-sectional images of the brain. Endoscopes could be used to reach glioblastoma tissues during OCT measurements. Different types of OCT endoscopes have been described, integrating side-viewing and forward viewing probes, different scanning mechanism, or being combined with other imaging modalities (Gora et al., 2017). Compared with other imaging techniques, OCT equipment presents the advantages of being relatively cheap while producing images with high axial (1–10 μm) and temporal (10^−3^ s) resolutions without needing any contrast agent. A study has compared OTC images obtained from healthy and GBM human brain tissues extracted from patients. Lower optical attenuation was found in cancer than non-cancer tissues, suggesting that OCT could discriminate between healthy and tumor tissues during a surgical GBM treatment (Kut et al., 2015).

##### Magnetic imaging systems

**Intraoperative magnetic resonance imaging (iMRI)** can be divided in two categories. A first type of iMRI developed by IMRIS (IMRISneuro), Philips (Ingenia MR-OR), GE healthcare (MR surgical Suite), Odin Medical Technologies (PoleStar magnet), Medtronic Navigation (PoleStar), is directly conceived to be used as iMRI. A second category of iMRI, commercialized by Hitachi Medical System (AIRIS I and II), Siemens Medical Solutions (Magnetom Open Viva), and BrainLAB (BrainSuite), consists in MRI, which have been modified and adapted to be useable in the operation room (OR). iMRI is used to identify GBM cell location during a surgical operation. It can be subcategorized as iMRI of low field strength (0.12–1.5 T), enabling relatively easy and fast real time imaging but without a high resolution, and iMRI of high field strength (1.5–3 T) that are more difficult to use during surgery due to a longer acquisition time, but provide brain tumor images with enhanced resolution. iMRI has been shown to strengthen the safety of surgical procedure by imaging healthy tissues, hence preventing their removal, to increase the percentage of tumor resection, and possibly rather modest improvement in GBM patient survival (Coburger et al., 2017; Fukui et al., 2017; Khan et al., 2017).

**Functional Magnetic Resonance Imaging (fMRI)** are commercialized by large companies that already sell standard MRI equipment, such as Siemens (Siemens 3-T Trio fMRI), Philipps (Achieva 3.0T X-series scanner combined with the Eloquence system), GE Healthcare (BrainWave). fMRI enables to acquire blood oxygen level dependent (BOLD) MRI scans of the brain and hence to detect metabolic changes and abnormalities that are induced by changes in brain oxygen concentration. It complements standard MRI imaging, which only provides morphological information of the cerebral cortex. fMRI is used during brain surgery to detect parts of the brain that need to be kept in place such as those responsible for the production of speech or comprehension, which cannot be seen with standard MRI (Salama et al., 2018).

**Magnetoencephalography (MEG)** equipment, which is commercialized by companies such as Elekta (Elekta Neuromag TRIUX), is a functional neuroimaging technique that maps brain activity by recording magnetic fields produced by electrical currents occurring naturally in the brain. Since the strength of these magnetic fields is very low (~10^9^ lower than the earth magnetic field), it uses very sensitive magnetometers (SQUID sensors) to record them. MEG can be used during GBM surgery to identify locations of brain abnormalities using direct measurements of neuronal activity without necessitating full patient immobilization. However, the main drawback of this technique comes from the high cost of the MEG equipment, which does not seem to be widely used for GBM treatment (Szymanski et al., 2001).

**Navigated transcranial magnetic stimulation (nTMS)**, commercialized by companies such as Magstim (Rapid) or Nexstim (eXimia), delivers magnetic stimulation to spots on the motor cortex. The resulting electrical activity is monitored by electromyography (EMG). nTMS enables to obtain a map of the motor cortex area and hence to optimize tumor resection by preventing removal or damage of eloquent motor areas. It was also shown that the use of nTMS in GBM patients increases the rate of gross total resections by 17% (Frey et al., 2014).

##### Other imaging systems

**Diffusion tensor imaging fiber tracking (DTI-FT)**, developed by Brainlab (iPlanCranial), slicer (Slicer4), and Medical Analysis and Visualization (MedAlyVis), is a noninvasive imaging technique that measures the diffusion of water molecules in three dimensions within tissue through the application of multiple diffusion gradients. More specifically, it enables visualization of white matter tracts (WMT) often localized near glioma cells (Hana et al., 2014; Mickevicius et al., 2015).

**Intraoperative mass spectrometry (MS)**, uses an equipment such as Desi 2D developed by Prosolia, which is integrated in the operation room and delineate tumor regions by identifying and characterizing the mass and fragmentation patterns of the molecules involved in GBM at the nanometer scale (Pacholski and Winograd, 1999; Stoeckli et al., 2001; Agar et al., 2011).

### Treatments based on the application of an external source of energy

Apart from the radio-sensitizer KU-60019 that has only been tested on mice, GBM treatments using an external source of energy have been tested on humans.

#### Radiotherapy

In current radiotherapy treatments of GBM, patients are usually exposed to fractionated localized radiation using a standard dose of radiation of 60 Gy, delivered in 30–33 fractions of 1.8–2 Gy (Fuller et al., 2007). Radiotherapy treatments can be carried out using external or internal radiation sources, radioactive monoclonal antibodies, possibly using radio-sensitizer to enhance the effects of radiations.

##### External beam radiation therapy (EBRT)

EBRT is the most frequently method, which is used for administering radiation therapy (X-ray and protons essentially) to glioblastoma tumors. High energy rays or beams produced outside of the brain are orientated toward the tumor to cover the whole tumor volume (Mann et al., 2018).

**Three dimensional conformational radiation therapy (3D-RT) or Image guided radiation therapy (IGRT)** use Clinac, Radixact, or Synergy equipment, commercialized by Varian, Accuracy, and Elekta, respectively, that generate X-ray photons of typically 4–20 MV. In this treatment, the glioblastoma tumor is first imaged in 3D using CT, MRI, PET, or PET-CT scan, and a computer program then designs the orientation of the radiation beam applied on patient's head to cover the whole tumor volume while sparing healthy tissues. Patients are typically exposed to 50–90 Gy (Tanaka et al., 2005; MacDonald et al., 2007; Thibouw et al., 2017). In a clinical trial involving 184 GBM patients, survival at 5 years was shown to reach 51 and 15% following 3D-RT and non-conventional radiotherapy, respectively (Tanaka et al., 2005), indicating that 3D-RT increases patient's survival compared with non-conformational radiation therapy.

**Intensity modulated radiation therapy (IMRT)** uses Radixact (Accuracy), Infinity or Precise treatment system (Electa), Vitalbeam or Clinac (Varian). It is similar to 3D-RT or IGRT, but has the additional feature of allowing an adjustment of the strength of the radiation beam, depending on the targeted region of the glioblastoma tumor. Compared with 3D-RT or IGRT, IMRT enables to deliver higher radiation doses within a shorter period of time without any toxicity increase (Amelio et al., 2010; Burnet et al., 2014). Treatment typically involves daily sessions of 10–20 min during 6–8 weeks.

**Helical-tomography (HT)** uses a HT system, commercialized by Accuray for example. HT is a type of IMRT that uses computed tomography (CT) to guide the X-ray beam to the desired tumor location. HT produces a narrower beam than LINAC used in conventional IMRT. This beam is delivered while the patient is moving enabling to better target different tumor sites without the need for a pause between different patient positions. HT was reported to better spare organ at risks than LINAC during GBM radiation therapy (Miwa et al., 2008; Koca et al., 2014).

**Stereotactic radiosurgery (SRT)**, carried out by Apex or Versa HD (Elekta), Truebeam or ClinaciX system (Varian), Artiste solution, Oncor K or M Class, Primus (Siemens Healthineers), is a non-invasive treatment method that uses pencil-thin beams of X-ray radiation that are focused on GBM tumor. Patient's head may be inserted in a frame, an imaging technique such as CT or MRI is used to locate the tumor and deliver the energy at tumor location. Compared with IMRT, SRS presents the advantage of delivering X-ray energy within less sessions (<5) during 6–8 weeks, using higher doses of radiation during each session (Yanagihara et al., 2016).

**Gamma knife**, commercialized by Elekta for example, is a specific type of SRT. It delivers a large number of X-ray beams (>200) that are focused on the GBM tumor with the help of a computer. Gamma knife was reported to be a safe treatment option for patients diagnosed with recurrent GBM. In terms of efficacy, it yielded a median survival after tumor recurrence ranging from 13 to 26 months, which is not significantly better than with other types of radiotherapies. When it was combined with chemotherapy, improved survival may have been observed among GBM patients, but a phase III appears necessary to confirm this result (Elaimy et al., 2013).

**Cyber knife**, commercialized by Accuray for example, is another specific type of SRT. Compared with Gamma Knife, Cyber knife presents the advantages of not requiring a metal frame around patient's head, of letting the patient lie while the radiation system moves around its head, and of not needing the patient to be anesthetized. Although Cyber knife presents several interesting technological features, it actually seems to have led to GBM tumor appearance when it was used to treat a patient with brain arteriovenous malformation (Xhumari et al., 2015). The balance between anti and pro tumorigenic effects of Cyber knife and other radiotherapy equipment should therefore be carefully examined before starting the treatment.

**Proton radiation therapy (PRT)** is carried out with an equipment such as Radiance 330 commercialized by Pro Tom International that generates proton beams, which deliver energy of 70–250 MeV within the tumor location. Compared with X-rays, PRT induces less energy penetration in healthy tissues than X-rays. It enables to reach antitumor efficacy using a lower level of radiation than X-rays and to minimize the exposure of radiations to organs at risks such as the hippocampi, sub-ventricular zones, hearing and visual apparatus, and pituitary gland. Several clinical trials, carried out on GBM patients treated by proton therapy, reported that this therapy was well tolerated, but they did not firmly conclude in an improvement in patient survival, due to the too small number of treated patients (Galle et al., 2015; Adeberg et al., 2017). Proton therapy may be of specific interest in children, which are more affected by long-term effects of x-ray therapy than adults.

##### Internal radiation therapy (IRT) or brachytherapy (BT)

Brachytherapy uses a radioactive substance located near or in the GBM tumor to deliver radiation therapy (Barbarite et al., 2017). BT enables to reduce side effects including damages to healthy tissues by concentrating the radiation beam in the regions where the tumor is located or is most the likely to recur. The longest median overall survival following BT that have been reported so far are 28 and 16 months for patients with newly diagnosed and recurrent GBM, respectively. Initially, a radioactive material was directly inserted in the GBM tumor. To avoid that physicians are exposed to a too large quantity of radiations, the radioactive material can be inserted in a catheter connected to the tumor. BT can be divided between low-dose rate brachytherapy (LDR-BT) and high-dose rate brachytherapy (HDR-BT), delivering less or more than 30 cGy/h, respectively. LDR-BT leads to less side effects than HDR-BT and to better benefit/risk ratio, but takes a longer time and induces more patient discomfort than HDR-BT. The radioactive substance used in BT is usually either I-125 (Iodine-125) or 192-Ir (iridium 192).

**Gliasite**, initially developed by Hologic and currently commercialized by Isoray, consists of a balloon, which is positioned in or near the GBM tumor during surgery and is then filled with a radioactive material containing I-125 (Iotrex [sodium 3-(^125^I)-iodo-4-hydroxybenzenesulfonate]). Gliasite enables the delivery of radiation dose to areas that are most at risk of recurrence. A clinical study carried out on 24 patients suffering from recurrent GBM showed that the treatment was safe, but it did not conclude in improved patient survival compared with other types of radiotherapies (Chan et al., 2005).

##### Radioactive monoclonal antibodies

**Cotara**, developed by Peregrine Pharmaceuticals, is a ^131^I-labeled chimeric monoclonal antibody that was designed to diffuse to necrotic area of GBM tumor and to bind to specific antigens expressed in cells belonging to this part of the tumor (histone H1 complexed with deoxyribonucleic acid). Cotara should then deliver a cytotoxic dose of ^131^I radiation to the adjacent living GBM cells (Patel et al., 2005). Clinical trials (phase I, NCT00509301 and phase II, NCT00677716) were carried out leading to a 2 months improvement in survival among 40 GBM patients in 2011. However, this result was not confirmed in a phase III and the author is unaware of any further clinical developments using radioactive monoclonal antibody for GBM treatment since 2011.

##### Radiosensisitizer

Several molecules were reported to increase antitumor efficacy of radiation when they were present in the tumor during radiation.

**KU-60019**, under development by AstraZeneca, is a kinase inhibitor, which was shown to radiosensitize glioma cells both *in vitro* (Golding et al., 2012) and *in vivo* on mice bearing GBM tumors (Vecchio et al., 2014). In mice bearing GBM, treatment consisting in KU-60019 administration and radiation led to a 25 days increase in survival compared with radiation alone (Vecchio et al., 2014).

#### Electric field therapy

**Tumor treating fields (TTFields)**, commercialized by Novocure, uses Optune consisting in electrodes positioned on patient's head that generate low intensity electric fields alternating at a frequency of 200 kHz, which selectively block tumor cell division during mitosis by interrupting during metaphase and/or anaphase the spindle assembly unusually occurring in healthy cells (Mun et al., 2017). When U-118 glioma cells were treated with TTF combined with standard chemotherapeutic drugs (Paclitaxel, Doxorubicin, Cyclophosphamide), it resulted in the destruction of most living cells after 70 h of treatment, while the drugs or TTF alone only slowed down cancer cell proliferation, suggesting that TTF should be combined with another treatment modality to reach optimal efficacy (Kirson et al., 2008), Preclinically, rats bearing intracranial GBM were treated with TTF during 6 days, leading to smaller tumors for treated than untreated rats. Interestingly, this study underlines the necessity of applying TTF in several directions to yield antitumor efficacy (Kirson et al., 2007). The author is not aware of a study showing full disappearance of GBM tumors in mice/rats treated with TTF and it seems that this treatment went directly to clinical trials without such demonstration. The efficacy of TTF was assessed clinically on patients with recurrent or newly diagnosed GBM (Benson, 2018). In particular, in a phase III clinical study involving 466 patients (EF-11), the addition of TTFields to standard therapy was shown to increase median overall survival from 15.6 without TTF to 20.5 months with TTF, to improve patient quality of life, and to lower incidence of serious adverse events (Stupp et al., 2015). Optune seems to be one of the only recent treatments leading to a statistically significant improvement in survival for patients suffering from GBM. However, such improvement is relatively modest and implies a very large increase in treatment cost by an average of 185,476 euros per patient (Bernard-Arnoux et al., 2016).

#### Laser therapy

**Magnetic resonance guided laser-induced interstitial thermal therapy (MRgLITT)** has been developed by Monteris (Neuroblate and Visualase). In this treatment, Magnetic Resonance Imaging is first used to localize the GBM tumor, a laser beam is transmitted through fiberoptics toward the tumor region and the resulting thermal energy heats the tumor at an average temperature of 43°C. Thermography enables to monitor and adjust temperature changes during the treatment. The mechanisms by which heat induces tumor destruction remain poorly understood, but possibly involve protein denaturation, membrane dissolution, vessel sclerosis, and coagulative necrosis. MRgLITT can serve to destroy tumor parts located in regions of the brain that are difficult to access and would possibly lead to injury of adjacent functional structures if surgery was used. Visualase and Neuroblate systems operate at relatively similar wavelengths of 980 nm and 1,064 nm, respectively, and powers of 12–15 W. However, the Neuroblate system can more precisely adjust light diffusion in the tumor by using both diffusing and side scattering modes compared with Visualase that only operates with a diffusing mode (Lagman et al., 2017). Furthermore, the Visualase system has only rarely been used for GBM treatment, its main therapeutic target being epilepsy (Patel et al., 2016). A first clinical trial carried out on 10 patients with 15–40 mm GBM tumor resulted in tumor necrosis 24–48 h following Neuroblate treatment (NCT007472253) (Sloan et al., 2013). Another clinical trial on 34 GBM patients did not show any improvement in survival for patients treated with Neuroblate, but it underlined the importance of heat homogenous distribution to reach the best treatment outcome (Mohammadi et al., 2014). It was also shown on 20 patients suffering from GBM that the Neuroblate system could open the BBB between 1–2 and 4–6 weeks following MRgLITT treatment, and hence possibly favor the diffusion of drugs through the BBB during this lapse of time (NCT01851733) (Leuthardt et al., 2016).

#### Radiofrequency treatment (non-thermal and thermal)

Radiofrequency hyperthermia was carried out on GBM patients by inserting electrodes into GBM tumors using CT-guided stereotaxis and applying 13 MHz radiofrequency hyperthermia during 1 h, leading to: (i) an increase in tumor temperature that remained below 43°C, (ii) the destruction of the BBB enabling chemotherapeutic drugs to reach the tumor, (iii) an absence of side effects. The treatment led to 80% of necrotic tumor and to a decrease in tumor diameter. Further assessment of this treatment is however necessary to conclude about its efficacy on a larger number of patients (Sun et al., 2013).

A device generating ultralow radiofrequency without inducing heat (Nativis Voyager) was developed by the company Nativis. It is supposed to enhance tubulin polymerization and inhibit cell division (Butters et al., 2014). In a first clinical trial involving 14 patients suffering from GBM (NCT02296580), treatment with Nativis Voyager was reported to result in no serious adverse events and in a progression free disease among 2 patients (Barkhoudarian and Wayne, 2017).

#### Hyperthermia therapy with ultrasound

An ultrasound device approved by the FDA and commercialized by Insightec (Exablate Neuro) focuses ultrasound waves to GBM to heat and ablate these tumors. A first in man study carried out on a patient suffering from recurrent GBM demonstrated that high-power sonications could be applied on GBM with the help of MRI, yielding partial tumor ablation without adverse effects (Coluccia et al., 2014).

### Molecular targeting

#### Drugs targeting GBM at molecular levels used on humans

**Mibefradil**, under development by Cavion, is a drug that selectively blocks T-type channels, which are overexpressed in GBM tumors and are involved in angiogenesis and invasion of tumor cells. In a phase II study, Mibefradil was administered to 27 GBM patients. It was well tolerated and resulted in some responses, i.e., it increased overall survival (OS) and progression free survival (PFS) of GBM patients by 15 and 2 months, respectively (Holdhoff et al., 2017). However, efficacy needs to be confirmed on a larger cohort of patients.

**Temozolomide (TMZ)**, which is commercialized by Merck, is an alkylating agent that breaks DNA double-strand and also reduces the activity of a DNA repair enzyme, called O 6 methylguanine-DNA methyltransferase (MGMT), hence promoting GBM tumor cell death (Thomas et al., 2017). It is one of the only chemotherapeutic drugs, which has shown some clinical efficacy and is currently prescribed to treat GBM. It is used following radiotherapy treatment at a daily dose of 150–200 mg/m^2^ of body-surface area (BSA) for 5 days every 28-day cycle. In a large phase 3 clinical trial, the efficacy of a treatment using TMZ with concomitant radiation therapy followed by adjuvant TMZ for 6 months was shown to improve median overall survival (MOS) and 2-year survival by ~2 months and 16%, respectively compared with a treatment using only radiation (Stupp et al., 2005).

**Gliadel**, which is sold by MGI Pharma, is composed of wafers containing biodegradable polymers containing 3.85% carmustine that are placed in the resection cavity at the time of surgery for patients with primary or recurrent GBM. Carmustine is an alkylating agent of DNA and RNA. It has been shown to improve median survival of GBM patients by 2–4 months (Chaichana et al., 2011), and resulted in adverse effects that were significant but not superior to those observed with SOC (Perry et al., 2007).

**Val-083 (Dianhydrogalactitol)**, under development by Del Mar Pharmaceuticals, was reported to cross the BBB, to be absorbed more importantly in cancer than healthy cells, to bind to GBM cell DNA, leading to GBM cell death with more efficacy than other DNA drugs. VAL-083 was shown to be active against MGMT-unmethylated GBM cells which are resistant to treatment with TMZ and nitrosoureas. In a clinical trial, it increased GBM patient OS by 8 months (Eagan et al., 1979). For some reasons unknown to the author, despite of promising clinical efficacy, VAL-083 (dianhydrogalactitol) was not widely used since 1979 and seems to have been re-discovered only recently.

**Afatinib**, which is under development by Boehringer Ingelheim, is an irreversible inhibitor of epidermal growth factor receptor (EGFR), tyrosine kinase activity, and tumor cell proliferation (Taylor et al., 2012). In a phase I/II study, Afatinib was shown to have a manageable safety profile but resulted in limited activity among patients with recurrent GBM (Reardon et al., 2014a).

#### Drugs targeting GBM at molecular levels tested pre-clinically

**Aldoxorubicin**, developed by CytRx, contains doxorubicin, a well-known intercalating DNA agent, combined with a linker-molecule that specifically binds to albumin in the blood. Compared with Doxorubicin, Aldoxorubicin increases the amount of drug delivered while minimizing toxicity. When immune compromised mice bearing GBM tumors were treated with aldoxorubicin, the drug was observed to accumulate in the tumor and not in normal brain, to reduce the number of GBM dividing cells, and to lead to an OS of more than 63 days, compared with ~25 days for animals treated with doxorubicin or saline (Marrero et al., 2014).

**ANG-1005**, which is under development by Angiochem, consists of three molecules of paclitaxel conjugated to a peptide acting as a brain delivery vector (Angiopep-2), which improves penetration through the BBB by transcytosis (Bertrand et al., 2011). Once inside glioma tumors, paclitaxel is expected to prevent microtubule de-polymerization, and hence to inhibit tumor cell proliferation. Mice bearing U87 MG glioblastoma, which received ANG1005 at a dose of 50 mg/kg, were shown to live 3 days longer than untreated mice (Régina et al., 2008).

**CBL0137 (Curaxin)**, which is under development by Buffalo Biolabs and Incuron, is expected to trigger antitumor activity by binding to DNA and inactivating the Facilates Chromatin Transcription (FACT) complex, which repairs transcription and replication mechanisms of DNA. In mice bearing U87 GBM, the administration of 35–70 mg/kg of CBL037 and TMZ was shown to increase mouse maximal survival by 55 days compared with untreated mice (Barone et al., 2017).

### Anti-angiogenic

#### Anti-angiogenic GBM drugs used on humans

**Bevacizumab (BV, avastin)**, which is commercialized by Genentech for GBM treatment, is a human monoclonal antibody that inhibits vascular endothelial growth factor (VEGF), and has been approved for GBM treatment since 2009 in the USA. However, for patients suffering from GBM, the use of BV does not increase survival by more than 4 months in average and other benefits in terms of improved quality of life have not been demonstrated (Diaz et al., 2017). Studies combining the use of BV with other cytotoxic drugs have been published (Herlinger et al., 2016) or are currently ongoing to examine potential additional patient survival benefit with these combinations (Tamura et al., 2017).

**MLN518 (Tandutinib)**, which is under development by Millennium Pharmaceuticals, is an inhibitor of type III receptor tyrosine kinase (PDGF receptor-β, Fms-like tyrosine kinase 3, c-Kit). A first phase II study carried on patients receiving MLN518 with recurrent GBM was closed due to the lack of efficacy of the treatment (Batchelor et al., 2016). Another phase II clinical study investing the combination of BV with MLN518 for GBM treatment reported enhanced toxicity without improved efficacy compared with BV alone (Odia et al., 2016).

**Enzastaurin**, which is developed by Eli Lilly, is expected to specifically target and inhibit protein kinase C (PKC), hence preventing tumor growth and proliferation. Two phase II studies, which enrolled between 66 and 88 patients, did not show increased survival in patients receiving Enzastaurin compared with untreated patients (Kreisl et al., 2010; Butowski et al., 2011). In another phase II study on 81 GBM patients, Enzastaurin treatment combined with BV did not improve patient survival compared with treatment using BV alone (Odia et al., 2016). A phase III study carried out on 266 patients, which compared Enzastaurin and laumustine treatments, did not conclude in improved efficacy using Enzastaurin (Wick et al., 2010).

**AZD2171 (Cediranib)**, under development by AstraZeneca, is an anti-angiogenic drug that inhibits tyrosine kinase with activity against PDGF receptors and c-Kit. Preclinical studies carried out on mice bearing U87, U118, and CNS1 glioblastoma, which received Cediranib orally, showed that this drug did not affect tumor growth, but led to a slight increase in mouse survival by 5–10 days compared with untreated mice (Kamoun et al., 2009). In a phase III clinical study on 325 GBM patients, who were first treated by radiotherapy and TMZ chemotherapy, administration of AZD2171 alone or on combination with lomustine did not result in PFS improvement (Batchelor et al., 2013). A phase I study, in which GBM patients were treated with Cediranib and Cilengitide, also concluded in the absence of treatment efficacy (Gerstner et al., 2015).

#### Anti-angiogenic GBM drugs tested on animals

**Altiratinib (DCC-2701)**, which is under development by Deciphera Pharmaceuticals, is an anti VEGF drug that was designed to overcome BV resistance by targeting proto-oncogene MET, TIE2-expressing macrophages, and VEGFR2. In the GSC17 glioma xenograft model, administration of a combination of altiratinib and bevacizumab significantly prolonged survival compared with treatment using bevacizumab alone, suggesting that Altiratinib could be used to improve bevacizumab therapeutic efficacy (Piao et al., 2016).

**SapC-DOPS (Saposin, BXQ-350)**, which is under development by Bexion Pharmaceuticals, is made of SapC introduced in DOPS nano-vesicles. It is thought to trigger anti-tumor activity by targeting phosphatidylserine present in large quantity in the outer membrane of tumor associated vasculature and by preventing TGF-β expression and tumor coagulation (Blanco et al., 2015). Mice xeno-grafted with U87 glioma cells, which received intravenous injection of SapC-DOPS, displayed tumor growth delay compared with mice treated with DOPS (Blanco et al., 2015). In another study, mice bearing intracranial U87ΔEGFR-Luc and X12v2 glioma received intravenous injection of SapC-DOPS, resulting in full tumor disappearance 250–350 days following drug injection among 25–75% of treated mice (Wojton et al., 2013). Interestingly, SapC-DOPS could also be conjugated with Gd (Winter et al., 2015), or with iodine-127 or iodine-124-fluorescent markers (Blanco et al., 2016), to image GBM tumors.

**VB-111**, which is under development by VBL Therapeutics, consists of a non-replicating Adenovirus, which specifically targets endothelial cells within tumor vasculature (Gruslova et al., 2015). Rats and mice bearing U87MG and U251 tumors, respectively, which received a single dose of VB-111, were shown to live slightly longer (a few days) than untreated animals (Gruslova et al., 2015).

**TC-A237 (Alisertib)** is under development by Takeda Pharmaceuticals Internationals that bought Millenium, which originally filed the patent protecting Alisertib. Alisertib acts against the tumor by inhibiting Aurora-A kinase. Mice bearing GB169 or GB30 glioma xenografts received orally TC-A237, resulting in an increased maximum survival by 5–15 days (Van Brocklyn et al., 2014).

### Kinase inhibitor against GBM tested pre-clinically

**GDC-0084**, under development by Genentech and Kazia Therapeutics, is a brain penetrant inhibitor of PI3K and mTor. When it was orally administered to mice bearing U87 MG glioblastoma, it led to significant tumor volume decrease, but given the absence of survival curve in this study, it is difficult to conclude about the disappearance (or not) of the tumor (Heffron et al., 2016).

### Immunotherapies

Immunotherapy seems to be the therapeutic approach, which brings the most important amount of hope to yield efficient GBM treatment. It has therefore become the most studied one. The number of clinical trials testing immunotherapies against GBM has increased from 3 in 1999 to 9 in 2015 (Calinescu et al., 2015). At the same time, there has been a real surge in the number of publications related to this topic (from 15 in 1999 to 164 in 2017 according to pubmed). The reader is redirected toward the large number of excellent reviews on this topic (Calinescu et al., 2015; Binder et al., 2016; Desaia et al., 2016; Hodges et al., 2016; Kamran et al., 2016; Dunn-Pirio and Vlahovic, 2017; Farber et al., 2017; Lyon et al., 2017; McGranahan et al., 2017; Miyauchi and Tsirka, 2017; Sahebjam et al., 2017; Tivnan et al., 2017), providing details about current or past clinical trials (Binder et al., 2016), and the different modes of action of these treatments (Calinescu et al., 2015; Curry and Lim, 2015).

#### Active immunotherapy (vaccine) tested clinically

**Rindopepimut (Rintega, CDX-110)**, under development by Celldex, is a vaccine composed of peptides. It was designed to treat patients expressing a mutant of EGFR (EGFRvIII), which is present among 20–30% of GBM patients and is absent on healthy cells. Rindopepimut should therefore specifically target GBM cells. It operates by triggering humoral and cellular responses against EGFRvIII-positive cells (Babu and Adamson, 2012). Mice bearing B16-msEGFRvIII tumors were treated with antibodies acting against EGFRvIII (Y10) with antitumor mechanism equivalent to that of CDX-110. They displayed a maximal survival day, which was 100 days larger than untreated mice, but this improvement was only observed for intra-tumor injection. Intravenous (IV) injection failed to increase mouse survival (Sampson et al., 2000). At first, phases I and II clinical trials carried out on GBM patients vaccinated with Rindopepimut seemed to suggest larger progression-free and overall survival times on patients expressing EGFRvIII than on those missing EGFRvIII (Schuster et al., 2015). However, this result was not confirmed in a phase III clinical trial, carried out on 745 GBM patients expressing EGFRvIII, which were first treated by maximal surgical resection and chemo-radiation. Indeed, this trial led to an overall survival, which was similar at 20 months for patients treated with CDX-110 and TMZ and those receiving TMZ alone (Weller et al., 2017).

**SurVaxM**, under development by MimiVax, is a peptide vaccine that targets survivin, a protein responsible for glioma cell survival, which is present among 95% of GBM patients. A first in man study carried out on patients with recurrent GBM demonstrated the safety and immune response induced by the vaccine and suggested an apparent increase in PFS and OS by 8 months and 56 weeks, respectively, compared with patients receiving chemotherapy (Fenstermaker et al., 2016).

**Prophage (G-100, G-200, Vitespen)**, under development by Agenus, is a clinical vaccine containing a heat shock protein peptide complex (HSPPC-96), in particular the heat shock protein gp96. It is a patient specific vaccine fabricated using patient's tumor tissue. It is expected to trigger an anti-tumor immune response, possibly involving CD8^+^ and CD4^+^ T cells, as was observed during the prophage treatment of wild type Balb/c mice bearing fibrosarcoma tumors (Chakraborty et al., 2016). In a phase II GBM clinical trial, patients treated with Prophage and SOC (radiation and TMZ) displayed an increase in PFS and OS of 10 and 8 months, respectively, suggesting clinical efficacy. However, efficacy still needs to be confirmed on a larger cohort of patients (Chakraborty et al., 2016).

**Gliovac (ERC 1671)**, which is under development by Epitopoietic Research Corporation (ERC), is composed of autologous antigens, surgically removed from patient's tumor tissue, which are administered together with allogeneic antigens coming from glioma tissues resected from other GBM patients. A phase I study showed that 100% of patients treated with Gliovac were still alive 6 months following the beginning of treatment compared with only 33% for the controlled group (Schijns et al., 2015). This suggests clinical efficacy of Gliovac, which is currently further investigated in a larger clinical phase II (NCT01903330).

**IMA950**, which is under development by Immatics Biotechnologies, is an immunotherapeutic multiple-peptide vaccine, specifically developed to treat GBM. It contains tumor associated peptides (TUMAP) found on human leukocyte antigen (HLA) surface receptors coming from primary human GBM tissue. It is designed to activate cytotoxic T cells against tumor cells expressing TUMAP and also to prevent potential tumor escape mechanisms. A phase I clinical trial carried out on HLA-A*02 positive patients seems to have highlighted an anti-tumor immune response, but it did not conclude in any increased survival (Rampling et al., 2016). Further studies therefore seem necessary to examine the potential therapeutic benefit of this vaccine.

**DCVax-L**, which is under development by Northwest Biotherapeutics, seems to be the most advanced dendritic cell (DC) vaccine. It contains a combination of autologous tumor antigens with patient's own antigens. Following injection to the patient, DCVax-L should enable DC to present their surface tumor antigen to the CD4 and CD8 T cells and hence to activate these immune cells specifically against the tumor. A clinical phase I/II showed that patients treated with DCVax-L displayed OS and PFS, which were longer than those of the historical control by 21 and 15 months, respectively (Polyzoidis and Ashkan, 2014). A phase III is currently ongoing to further confirm (or not) a therapeutic benefit.

#### Passive immunotherapy (anti-body based) tested clinically

**Depatux-M (ABT-414)**, which is under development by AbbVie, is an antibody-drug conjugate that preferentially binds to EGFR, which is overexpressed in glioma cell and present in 50% of GBM patients. It then internalizes in cancer cells where it releases an anti-microtubule agent, called monomethyl auristatin F, MMAF, triggering tumor cell death. In a phase I clinical trial, ocular toxicity was observed and it is too early to conclude about any clinical efficacy of Depatux-M (Van den Bent et al., 2017).

**Asunercept (APG101, CAN-008)**, which is under development by Apogenix, is designed to block CD95 pathway by inhibiting CD95 ligand, which consists of the CD95 receptor extracellular domain fused to the Fc domain of IgG. A phase II clinical trial carried out on 91 patients suffering from recurrent GBM showed that APG101 administration combined with radiotherapy increases patient PFS and PFS6 by 2 and 17%, respectively, compared with radiotherapy alone (trial: NCT01071837). This suggests that APG101 leads to survival benefit, but this result still needs to be confirmed on a larger cohort of patients within a phase III clinical trial (Wick et al., 2014).

**MEDI-3617 and MEDI-575**, which are under development by MedImmune, are novel anti-PDGFRα antibodies. In mice bearing GL261 or U87 tumors, MEDI1317 was shown to increase mouse survival only when it was combined with cediranib (Peterson et al., 2015). In a phase II clinical study involving 56 patients with recurrent GBM, the administration of MEDI-575 was shown to be well tolerated but did not result in any significant clinical activity (Phuphanich et al., 2017).

#### Passive immunotherapy (check point inhibitor) tested pre-clinically

**NOX-A12**, which is under development by Noxxon Phama AG, is an anticancer agent that neutralizes CXCL12 blocking CXCL12 signaling through its two receptors, CXCR4 and CXCR7. Rats bearing brain tumors induced by injection of carcinogen ENU had a maximal survival, which was up to 150 days longer when they were injected with NOX-A12 compared with untreated rats (Liu et al., 2014). Mice bearing G12 glioma tumors, which were treated with a combination of bevacizumab and NOX-A12, were shown to live ~15 days longer than those treated with NOX-A12 alone (Deng et al., 2017).

### Nanotherapies

#### Nano-therapy tested on human

**Nanocell**, which is under development by EnGeneIC, is composed of a minicell containing doxorubicin, which is conjugated with bi-specific proteins that target EGFR overexpressed in glioma cells. In a first in man study, signs of toxicity were not reported but efficacy has not yet been assessed (Whittle et al., 2015).

#### Nano-therapy tested *in vitro*

**Gold Nanoparticles**, which are under development by Midatech Pharma, are 2 nm Au NPs, coated with sugar moieties and/or thiol-polyethylene glycol-amine (PEG-amine). They were shown to be chemo-radiosensitisers, i.e., to enhance the antitumor efficacy generated both by X-rays and chemotherapy *in vitro* (Grellet et al., 2017).

### miRNA targeting GBM drug tested on humans

**TargoMiR**, under development by EnGeneIC, are micelles filled with miR-16, which target EGFR and are designed to counteract the loss of the miR-15 and miR-16 miRNA family, which is associated with tumor growth. First clinical results were reported for the treatment for the treatment of mesothelioma, but not yet for glioblastoma (Van Zandwijk et al., 2017).

### Glioma stem cell targeting drug tested clinically

**ICT-107**, under development by ImmunoCellular Therapeutics, is an autologous dendritic cell vaccine pulsed with class I peptide from tumor-associated antigens (TAA) designed to target six different tumor associated antigens (TAA). A clinical study carried out on 21 GBM patients has reported larger PFS and OS in patients with increased expression of TAA as well as a decrease or absence of CD133 overexpressed on glioma stem cells in 5 patients following a second resection (Phupahnich et al., 2013).

### Gene therapy against GBM tested clinically

**TOCA511 combined with TOCAFC**, which is under development by Tocagen, is a retroviral replicating vector (RRV), which leads to the permanent integration of RRV into the cancer cell genome, and encodes yeast cytosine deaminase, which further converts the antifungal prodrug 5-fluorocytosine (FC) into the anticancer drug 5-fluorouracil, hence mediating local tumor destruction. In mice bearing U87, Tu-2449, TOCA injection seemed to have resulted in tumor disappearance among 40–100% of treated mice, depending on tumor type, quantity of drug injected, and the combination (or not) of TOCA511 with TOCAFC (Ostertag et al., 2012; Huang et al., 2015; Yagiz et al., 2016; Hiraoka et al., 2017; Mitchell et al., 2017). The combination of TOCA511 and TOCAFC treatment was also tested in a phase I clinical trial on patient suffering from GBM, resulting in favorable safety profile and better OS compared with lomustine treatment (Strebe et al., 2016).

### Virus as GBM treatment tested clinically

**ParvOryx (H-1PV)**, which is under development by Oryx GmBH, is an oncolytic virus designed to specifically target and destroy cancer cells. A phase I/IIa clinical trial carried out on GBM receiving H-1PV showed that H-1PV was well tolerated, crossed the BBB, spread through the tumor, and possibly triggered an antitumor immune response through antibody formation and specific T cell response. Patient survival seemed to have been prolonged, but a phase III clinical trial is necessary to confirm this result (Geletneky et al., 2017).

## Early diagnosis

The symptoms associated with GBM include headache, seizure, memory losses, personality changes, motor weakness, visual symptoms, language deficit, increased intracranial pressure leading to nausea, vomiting, and cognitive impairment (Kondziolka et al., 1987; Chang et al., 2005). These symptoms often appear when GBM tumor is already quite large and difficult to treat. It therefore seems important to develop diagnosis methods that can detect GBM before the appearance of any symptom. By contrast to other cancers for which early detection is carried out on a regular basis over a large percentage of the population at risk, for example by using mammography for breast cancer or prostate specific antigen detection for prostate cancer, GBM is not currently screened in this fashion. Physical examinations can diagnose GBM by detecting focal, visual field, and cognitive impairments, but these symptoms are usually detected when the extent of healthy tissue destruction is already quite significant. Standard imaging techniques such as MRI, CT, and PET, are costly and possibly lack the sensitivity to detect GBM tumors of small sizes. Therefore, their regular use to screen the whole population has not yet been considered. To detect GBM, stereotactic biopsies require knowing precisely where the tumor is located and could be used to confirm the presence (or not) of GBM but with more difficulty for initial GBM detection. Other diagnosis methods are under development to detect GBM biomarkers at a molecular level, but the author is not aware of any breakthrough in this field and more efforts should probably be spent to develop new methods for early GBM detection.

## Preclinical models

To carry out a successful clinical trial on GBM patients leading to significant efficacy, it seems essential to have first optimized the treatment pre-clinically. However, studies on animals bearing GBM cannot easily be performed for the following reasons. First, governmental regulations on animal experimentations have become more and more stringent and restrictive (Workman et al., 2010). Second, GBM animal models are prone to a series of drawbacks such as too small GBM tumors in mice and rats, cell-line xeno-grafts leading to tumors being genetically different from a human GBM, patients derived GBM (PDX) growing with difficulty and yielding tumor inhomogeneity, human GBM being only grown on immune-deficient mice lacking full immune system, animal GBM reported to be different from human ones, large animals with naturally occurring GBM such as dogs being scarce and treated at a cost and level of sophistication approaching those met in a human. To overcome these drawbacks, it therefore seems necessary to test GBM treatments on several different animal models described in more details below.

### Small animals

Current preclinical mouse glioblastoma models are divided between xenografts (cell-line and patient derived) and genetically engineered models.

### Mice

- **Glioblastoma cell line xenografts**, such as the commercially available GBM immortalized cell lines U87, U251, T98G, and A172, are usually relatively easy to grow, but these cell lines are reported to be quite different from a GBM of a human patient, being circumscribed, having different genotype (Huszthy et al., 2012), MHC and integrin expression (Huszthy et al., 2012), as well as lacking certain GBM features such as single-cell invasion, tumor necrosis, or microvascular proliferation (Mahesparan et al., 2003). Furthermore, they can usually only be xeno-grafted into immune-deficient mice such as nude, NOD/SCID, and NOD/SCID gamma mice, with a weakened immune system that cannot be fully activated against the tumor. Furthermore, the differences between cell line xenografts and human GBM should be taken into consideration for the development of a molecular targeting GBM treatment in which the GBM composition is essential. However, when the mechanism of antitumor activity involves the application of radiation (X-ray, proton, laser, magnetic field) and is of physical origin, the treatment may act relatively similarly on xeno-graft cell line than on other GBM models (Alphandéry et al., 2017a,b; Le Fèvre et al., 2017).
- **Patient-derived xenografts (PDX)** are GBM tumors grown orthotopically or subcutaneously on mice by administering either biopsied patient tumor tissue (Fei et al., 2010; Kim et al., 2016), or cultured tumor spheres (Kang et al., 2015). Compared with GBM cell line xenografts, PDX present the advantage of reflecting the genetic and histological features of patient's GBM tumor, in particular being prone to single-cell invasions and tumor angiogenesis (Wakimoto et al., 2011). However, PDX have also been associated to the following drawbacks: (i) only 10–20% of PDX can successfully be grown on mice (Huszthy et al., 2012), (ii) PDX can be relatively inhomogeneous, (iii) PDX are usually grown on immune-deficient mice and therefore do not fully reflect patient's antitumor immunity. Despite of these weaknesses, it was demonstrated that PDX generated from cultivated patient-derived GBM stem cells (neurosphere) could better represent the GBM of a patient than immortalized GBM cell lines (Patrizii et al., 2018). The reason for introducing PDX cell lines also comes from the fact that a number of studies reported antitumor efficacy using immortalized GBM cell lines without demonstrating antitumor efficacy on humans (Patrizii et al., 2018).
- **GBM genetically engineered mouse (GEM)** models involve mice in which certain genes have been inactivated to study genetic alterations involved in GBM tumor initiation and progression. Although GEM models can help understanding the role of tumor microenvironment (Charles and Holland, 2010), they yield different tumors from human GBM, and tumor growth cannot easily be controlled in GEM.
- **Syngenic mouse models** include chemically induced (GL261, GL26, CT-2A) or spontaneous (P560) GBM mouse models (Oh et al., 2014). These models use immune-competent mice and are thus suitable for analyzing potential anti-tumor activity of GBM drugs. However, it remains uncertain whether these GBM animal models truly represent human GBM.

### Rats

Compared with mice, rats enable the growth of larger GBM tumors, which can be advantageous for the development of certain GBM treatments. However, these tumors are not genetically engineered and targeting of specific pathways associated with GBM can therefore not be studied with rats. Most frequent GBM rat models include:

- **C6 glial tumors** were originally produced 8 months following injection of MNU to rats. These tumors contain certain features of human GBM such as the presence of pleomorphic cells, tumor invasion into the surrounding brain, expression of genes involved in human GBM, such as PDGFb, EGFR, IGF-1, and Erb3 (Morford et al., 1997; Guo et al., 2003).
- **9L gliosarcoma**, originally grown on rats and collected 6–7 months after MNU administration, were used to develop GBM drugs, in particular drug transportation across the BBB (Khan et al., 2005) as well as MRI and PET imaging techniques (Bansal et al., 2008). They also possess common properties with human GBM such as mutated p53, overexpressed EGFR, the presence of cancer stem cell (CSC), and a certain level of immunogenicity when they are grown in Fisher rats (Barth and Kaur, 2009).
- **T9 rat glioma** is similar to 9L gliosarcoma (Barth, 1998).
- **CNS-1 glioma**, originally produced by repeated MNU injections during 6 months, formed tumors with many common features with those of human GBM such as invasive growth, nuclear atypia, necrotic foci, macrophages, and T cells infiltration in the GBM tumor (Owens et al., 1998; Matthews et al., 2000; Nutt et al., 2001; Candolfi et al., 2007).
- **RG2 and F98 glioma**, originally produced by injection of ENU in rats, are highly invasive and overexpress PDGFb, Ras, and EGFR, representing well some of the behaviors of a human GBM (Weizsäcker et al., 1982). However, both tumors appear to be less immunogenic than human GBM (Tzeng et al., 1991).
- **BT4C glioma** initially developed by administrating ENU to pregnant rats, are characterized by dilated, non-uniform blood vessels, irregular nuclei, areas of high and dense cell proliferation (Stuhr et al., 2007), the presence in tumor periphery of a larger number of VEGF, tPA, uPA, and larger micro-vessel density (Barth and Kaur, 2009). This cell line was used to study the combination of VEGF inhibition with temozolomide and radiation (Sandström et al., 2008).
- **RT-2 glioma** was developed differently from the previously described cell lines, i.e., not through carcinogen exposure but using intracranial injection of Rous sarcoma virus in rats (Copeland et al., 1976). These tumors, which trigger a CD8+ immune response, may be used to study cancer immunotherapy.
- **A transgenic rat model** was developed by using the S100b promoter that led to the expression of a viral form of EGFR (v-erbB) (Ohgaki and Kleihues, 2005) and to the appearance of malignant glioma among a small portion of treated rats (Ohgaki and Kleihues, 2005; Yokoo et al., 2008). Although this model could be used, in particular to study glioma infiltration by tumor-associated macrophages (Sasaki, 2017), it requires further optimization to yield a larger percentage of rats with GBM.

### Large animals (Dogs)

In some respects, the dog GBM model appears more suitable for preclinical drug screening than the mouse or rat model. Indeed, the size of a dog tumor is closer to that of a human GBM. Furthermore, dog GBM models are possibly more representative of human GBM, with TP53, EGFR, PDGFRα, and IGFBP2 GBM markers being overexpressed in dog GBM, (Higgins et al., 2010), as well as cancer stem cell (CSC) and associated CD133 being present in dog GBM (Stoica et al., 2009). However, GBM are scarce in dogs with an incidence rate of only 7 per 100,000 dogs (Dobson et al., 2002). Animal experimentation on dogs also relies on the owner consent and leads to higher cost and more ethical issues than mouse or rat studies (Hansen and Khanna, 2004). GBM dog models were used to examine the efficacy of several GBM treatments such as: (i) immunotherapy by implanting stimulated autologous lymphocytes into the tumor bed (Ingram et al., 1990), (ii) brachytherapy by inserting an inflatable balloon (Iotrex) containing iodine-125 in the GBM resection cavity (Stubbs et al., 2002), (iii) gene therapy by administering a recombinant adenovirus to dogs, (iv) increased quantity of administered drug by using convection-enhanced delivery (CED) under the application of a pressure gradient (Dickinson et al., 2008).

## Delivery of the treatment

In order to reach efficient antitumor activity against GBM, treatments relying on physical and chemical mechanisms should both be improved.

Physical methods of GBM destruction, which rely on the use of previously described surgery, radiotherapy, lasers, or electric fields, combined with imaging, would most likely need to be sufficiently precise to image and remove GBM cells at the single cell level. This is not yet possible in the clinic, not only due to a lack of sensitivity of the current imaging and surgical tools, but also to part of GBM cells being located in difficult to access regions of the brain. Even if this became possible technologically in the future, debris of tumor cells, genetically modified DNA, RNA, or other tumorigenic biological material, could remain in the organism after treatment and trigger tumor re-growth. It therefore appears that these physical methods, which are essential to remove the large majority of the GBM tumor, should be combined with other therapeutic approaches acting at a more molecular level.

Most chemical treatments against GBM present the advantage of being specific, *i.e*. they target a specific part of the tumor, which is present or expressed in larger quantity in the tumor than in healthy tissues. Such targets include A2B5, CD15, CD44, CD71, CD90, CD133, Integrin-α6, L1CAM (Xu et al., 2015; Glaser et al., 2017), miRNA (Kim et al., 2016), EGFR, PDGFR, BCR-Abl, FLT3, VEGFR, P13K, mTor, Ras/Raf/MAPK, microtubule inhibitor, topoisomerase inhibitor (Laquintana et al., 2009; Oberoi et al., 2016), telomere repeat-binding factor 2 (TRF_2_), MiR-21, MiR-125b, MiR-181, integrin such as αvβ_3_ and αvβ_5_ (Xu et al., 2015), tumor associated antigens (Platten et al., 2016), glioma stem cells (Hide et al., 2013). Although several GBM drugs have been shown to be able to interact with these targets leading to antitumor activity *in vitro* and/or in animals (Blanco et al., 2014; Paff et al., 2014; Thaci et al., 2014; Yang et al., 2015), most of them have not led to clear therapeutic benefit (Staedtke et al., 2016). This may be due to GBM drugs not efficiently reaching the tumor in humans, requiring GBM drug delivery to be improved to expect significant therapeutic activity on humans. The different routes of administration are described below.

- Oral is the easiest and most common route of administration, used for Mibefradil, TMZ, Curaxin, Altiratinib, MLN518, Enzastaurin, AZD2171, GDC-0084, and TC-A237. While several of these drugs (Curaxin, Barone et al., 2017, Altiratinib, Smith et al., 2015, GDC-0084, Salphati et al., 2016, TMZ, Agarwala and Kirkwood, 2000) were reported to cross the blood brain barrier, other ones were observed to be blocked by the BBB (MLN518, Oberoi et al., 2016, AZD2171, Oberoi et al., 2016) due to the presence of BBB efflux transporters. A clinical study compared TMZ oral and intravenous administrations, concluding that both routes lead to a similar level of drug exposure (Diez et al., 2010).
- Intravenous/intra-arterial route was used for Bevacizumab, ANG-1005, SapC-DOPS, and VB-11 administrations. These drugs crossed the BBB in different ways, i.e. by disruption of the BBB with mannitol for Bevacizumab (Boockvar et al., 2011; Burkhardt et al., 2012), through the low density lipoprotein receptor-related protein 1 (LRP-1) pathway for ANG-1005 (Bertrand et al., 2011), by binding to anionic phospholipid phosphatidylserine (PtdSer) for SapC-DOPS (Wojton et al., 2013). For BCNU, intra-arterial administration was reported to yield 50 times more drug in tumor tissue compared with intravenous injection (Tyler et al., 1986), indicating that this administration route may lead to a larger quantity of drugs in GBM tumor than intravenous injection. Following treatment, intra-arterial delivery may also enable the neutralization with an antidote or removal by hemo-perfusion of drugs in excess (Dedrick et al., 1984; Oldfield et al., 1985), which could otherwise potentially yield side effects.
- Intradermal route essentially used to administer vaccine such as CDX-110, Gliovac, IMA950, DCVax-L, or ICT-107. This mode of administration is chosen for vaccine since the dermis and epidermis of human skin are rich in antigen-presenting cells, suggesting that it could favor an immune response (Hickling et al., 2011).
- Intratumoral route used for Gliadel and Panobinostat administrations. Intratumor administration presents the advantage of overcoming the problem of BBB penetration by enabling drug injection beyond the BBB, in or near GBM tumor cells. On the one hand, Gliadel, which is made of a chemotherapeutic drug (BCNU) embedded in a biodegradable co-polymer formed of 1,3-bis-(p-carboxyphenoxy)propane (pCPP) and sebacic acid (SA), is implanted in GBM resection cavity and progressively releases BCNU in the tumor (Bregy et al., 2013). Although Gliadel led to signs of efficacy (Bregy et al., 2013), they were accompanied by side effects including seizures and cerebral edema (Bregy et al., 2013). Therefore, the method of inserting drugs directly in the resection cavity requires further improvements to yield a better control on drug diffusion. On the other hand, Panobinostat is administered using another intratumor injection method under active development called convection-enhanced delivery (CED). In CED, the solution containing the drugs is pushed under pressure with a pump through one or several catheter(s) directly connected to the tumor. Advantages of CED come from the precise knowledge of the location where the drug is administered, the control over drug diffusion by adjusting the pressure with the pump, which enables interstitial delivery, the absence of injury caused by the catheters. CED was tested in a series of different clinical trials, leading to an acceptable safety profiles without however demonstrating any improved therapeutic efficacy (Vogelbaum and Aghi, 2015). CED therefore seems to require further refinement to be of added value for a GBM treatment.

## Accelerated program for GBM drugs to reach the clinic/market (orphan drug status)

An orphan status is given to a drug indicated for a rare disease, i.e., with an incidence lower than 5–7 per 10,000. Due to the relatively low incidence of GBM (5 per 100,000), GBM drugs are eligible to this status and most previously described drugs were given the orphan status by the regulatory agencies of various countries, most frequently by the EMA in Europe and FDA in the USA. This status was originally set up to encourage companies to develop treatments for rare diseases such as GBM for which the chances of generating a profit are undermined by the limited number of patients. Financially, it can provide: (i) partial coverage of clinical trial cost through tax credit reimbursement (50% in the USA and Japan, various percentages in Europe depending on the country), (ii) grants through programs that specially support orphan drug development (FDA and NHI in the USA, H2020 in Europe, NIBIO and AMED in Japan), (iii) discounts on regulatory fees necessary to obtain market authorization in USA, Europe, and Japan. In some countries like Japan, medical expenses can be covered by National Health Insurance in exchange of a control over drug price, a good system that enables both to lower drug development cost and to reach a reasonable drug selling price. Most importantly, the FDA and EMA grant a 7–10 years marketing exclusivity to an orphan drug in the USA and Europe, respectively, by not authorizing similar products to be commercialized during this lapse of time. The orphan status can also give access to scientific advice, which is provided by regulatory agencies to determine the right path toward clinical trials and commercialization and to avoid unnecessary costly and lengthy developments. Finally, it can lead to accelerated drug assessment and approval, which appear essential both to reduce drug development cost and to accelerate treatment access for GBM patients (Mariz et al., 2016). This status has been of enormous help to the pharmaceutical industry and it is uncertain that there would have been so many attempts to develop GBM treatments without it. However, it mainly relies on the seldomness of a disease. Indeed, among all drugs that received an orphan status by the EMA in 2010, Torisel reached the highest prevalence of 35 per 100,000 for the treatment of renal cell carcinoma (The Committee for Orphan Medicinal Products the European Medicines Agency Scientific Secretariat et al, 2011). The difficulty to develop a treatment and the severity of the targeted disease are two other essential criteria that should most probably be taken into consideration to maintain the orphan drug status to GBM drugs if/when GBM incidence increases in the future. The orphan status is also reserved to drugs and excludes medical devices. However, some medical devices, for example those of class III that are injectable and nano-formulated, may also deserve this status. This could ease the interactions between the pharmaceutical companies fabricating them and the regulatory agencies. An international authority could also be set-up to specifically manage/define the orphan drug status, enabling more uniform regulation and easier understanding of the implications of this status in the various regions of the world.

## Intellectual property

Concerning the field of tumor destruction by radiations such as X-ray, electric fields, or lasers, patents relate to different methods to image and then irradiate locally the tumor, to position the patient in the radiation field, to orientate and apply the beams toward the tumor, to measure and deliver the dose that can destroy the tumor while sparing healthy tissue, to produce a robotized irradiation system. With regard to surgery, we have identified patents on the Neuroarm robotic surgery system that allows to locally and precisely carry out tumor surgery while reducing the burden of tiring tasks for the surgeon. Anti-tumor drugs have been protected through various methods for targeting certain specific cellular receptors such as EGFR or CD95, various drugs compositions or methods for drug production, formulation, or administration, various systems of drug transports through the BBB, inhibitors of protein kinase, antitumor vaccine comprising dendritic cells activated against the tumor, various immunogenic compositions containing for example heat chock proteins.

**Figure 3:**
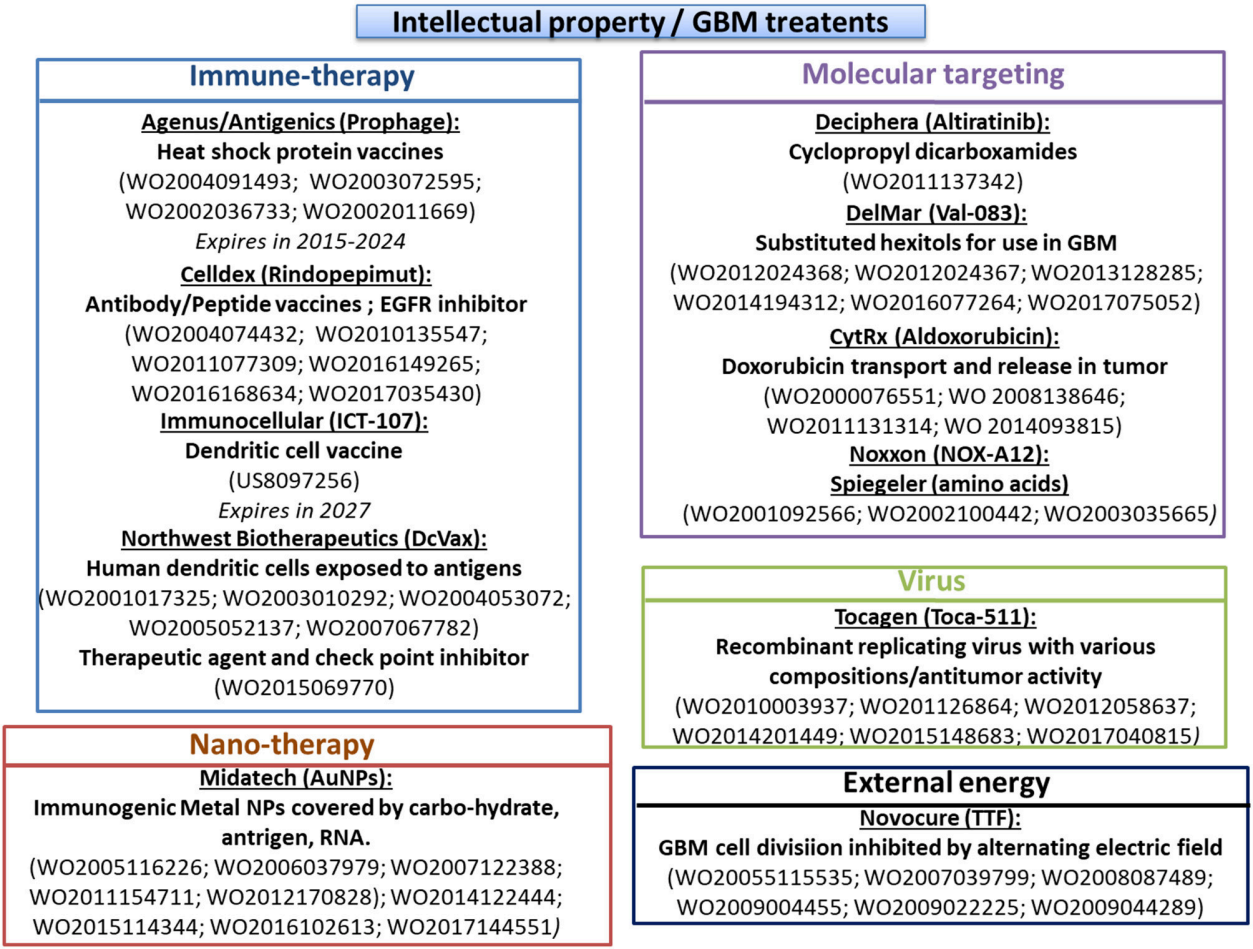
Patents submitted by the various companies developing or commercializing GBM treatments. On the one hand, since the GBM treatment name is often not mentioned in patents, it is possible that the number of patents is underestimated in some cases. On the other hand, since some of the listed patents have a broad scope, it may happen that they only partly cover the field of a specific GBM treatment, possibly leading to an overestimate in the number of patents in some cases.

Next, some of the features of the patent landscape in the field of GBM treatment are underlined. The distribution in number of patents earned by companies to protect their therapy is presented in Figure 3. It shows a discrepancy between companies possessing a large number of patents that may be able to develop their activity independently and those that earn only one or even no patent and may therefore have to seek additional protections or to negotiate patent license agreements with other structures. Secondly, two relatively old and well-established drugs (Avastin and TMZ) are still the subject of intense patent filing, due to their status of already approved drug and to their modest efficacy against GBM. In this case, the strategy to seek additional and more extended in time protection on these compounds essentially consists in filing patents on combinatory treatments including them and on various new methods to prepare/administer/use them for cancer treatment.

A detailed but non-exhaustive list of patents that is representative of the different domains in which protection has been sought for is presented below.

- **3D-RT**: imaging of the irradiated region using various methods such as CT, PET, MRI, HIFU, video (WO1989008430, WO1991000057, WO2004047923, WO2008120117, WO2010109585, WO2010109586, WO2012119649, WO20130679), equipment for positioning the patient during RT (WO2005122993), determination of the dose that needs to be used during RT (WO2007084272, WO2011005862, WO2012129661, WO2015042727, WO2016066590, WO2016070721, WO2016081916, WO2017105024), robotic system to determine radiation beam trajectory during RT (WO2010120534), system to avoid collisions during treatment (WO2015017630), set-up of a quality assurance system to enable reproducibility of treatment parameters (WO2015044781), equipment to generate beams in several direction with modulated intensity (WO2015062093).
- **Cyber-knife:** Radiation equipment (WO199200644, WO2005000102), methods to orientate the radiation beam toward the tumor including being in some cases a robotized (WO2000054689, WO2002019908, WO2004044612, WO2006130771, WO2010030463, WO2011109668), system including in some cases a robotic arm for positioning patient (WO2005039472, WO2005099819, WO2006124434), linear accelerator (LINAC) including in some cases a robotic arm coupled to the LINAC (WO2009005556, WO2010085723), method for determining the volume to be irradiated and/or dose of radiation and/or treatment parameters (WO2006130862, WO2006120863, WO20070386062, WO2007117650, WO2008002374, WO2008005129, WO2008005132, WO2010065740), imaging methods and apparatus to irradiate tumor region (WO2007005445, WO2009114859, WO2010030397, WO2011156526), radiation system with a gantry to image and guide radiotherapy (WO2011106433, WO2012099747).
- **Gamma-knife:** method for collimation of radiation beams (WO1996019262), apparatus for positioning the patient (WO1997017896), method for determining radiation dose (WO1998057705), X-ray/gamma ray radiation apparatus with/without linear accelerator with/without collimator with/without imaging system (WO1999034866, WO1999040759, WO2001011928, WO2001011929, WO2001013907, WO2002031837, WO2002049044, WO2003008986, WO200500498, WO2000018538, WO2005058419, WO2006013325, WO2006097274, WO2008141667, WO2009052845, WO2009056151, WO2009129817, WO2010006630, WO2010012983), surface mountable apparatus for combining radiation and imaging systems (WO2001012066), stereotactic apparatus for guiding radiotherapy (WO2001021085), method for controlling/direction radiation beams (WO2005051215), method for treatment planning (WO20091182021, WO2017109680), method for fixing patient's head during radiotherapy (WO2009129847), method to enable patient movement during radiation (WO2009137010), method for measuring radiation (WO2010031452).
- **IMRT:** Methods for treatment planning (WO2003099380, WO2011154853), apparatus for sequential generation of modulated beams (WO2004087254, WO2004098712, WO2015062093, WO2017070433), dose determination for IRMT (WO2005052721), method for focusing several beams during IRMT (WO2015176265), support system for patients (WO2009033035), IMRT combined with VMAT (WO2011042819).
- **Stereotactic radiosurgery:** Apparatus for SRS (WO1989005171, WO1994023663, WO1996041349, WO1997035641, WO2001076480, WO2017134582), laser or other marker for aligning SRS beam (WO1996039228, WO2016162784), dose estimate for SRS (WO1990014129, WO2005052721), patient positioning device for SRS (WO2014066108, WO2015030379), collision prevention system for SRS (WO2017007165).
- **Optune:** Method for treating a tumor with an electric field oscillating at different frequencies alone or in combination with other treatments such as photodynamic therapy (WO2005115535, WO2007039799, WO2008087489, WO2009044289) for treating tumor cells.
- **Neuroblate/Visualase:** MRI guided surgical apparatus that includes a laser that heats the tumor (WO2003051217).
- **Neuroarm:** Robot for brain surgery (WO2009037576, WO2009040677, WO2009044287).
- **ABT-414:** Composition comprising antibody against Epidermal Growth factor receptor (EGFR) that inhibits Bcl-xL (WO2017214282, WO2017214301, WO2015143382, WO2017214233); composition comprising antibody drug conjugates with specific drug/antibody ratio (WO2014152199).
- **Afatinib:** Preparation of various forms/compositions of Afatinib or Afatinib di-maleate (WO201221174, WO2013052157, WO2015007206, WO2015103456, WO2015186065, WO2016001844, WO2016027243, WO2016051380, WO2016079313, WO2016199076, WO2017033107, WO2017064039, WO2017093789, WO2017141271), use of Afatinib for cancer treatment (WO2015144934, WO2015153866, WO2016023822, WO2016027243).
- **Aldoxorubicin:** System of transport of a drug, in which a protein attached to the drug targets a tumor and specifically releases the drug in the tumor under pH changes, (US738777, WO2011131314), various formulations of doxorubicin (WO2008138646, WO201409381).
- **Altiratinib:** various kinase inhibitors (WO2007008917, WO2008033999, WO2013134298), derivatives of cyclopropane/cycloproply amides (WO2010051373, WO2011137342), pyridine/pyridine/pyrimidines derivatives (W02011139891, WO2013078295, WO2013134243, WO2013134252, WO2014145025, WO2014145028, WO2014145029, WO2015069266, WO20160661228, WO2014145004), imidazoline derivatives (WO2014145015), triazol derivative (WO2014145023), with anti-proliferative activity.
- **ANG-1005:** Pharmaceutical composition comprising aprotinin fragments Angiopep-1, Angiopep-2, conjugated (or not) to other compounds such as iduronate-2-sulfatase combined (or not) with lysomal enzyme, where this complex can cross the BBB and in some conditions accumulate in lysosomes (WO2007009229, WO2010142035, WO2013078562, WO2013078564, WO201385235, WO2014194427, WO2014194428, WO2016090495), paclitaxel, and a tonicity, buffering, bulking, solubilizing agent (WO2009127072).
- **Asunercept:** Cancer treatment with an inhibitor of CD95/CD95L in combination (or not) with an immunotherapeutic agent (WO2015107105, WO2015165973, WO2015197874, WO2017009429, WO2017051002).
- **Au NPs:** Metal nanoparticles with various ligands, mainly immunogenic ones (WO2005116226, WO2006037979, WO2007122388, WO2011154711, WO2012170828, WO2013034726, WO2013034741, WO2014122444, WO2014125256, WO2014135840, WO2015114341, WO2016162495).
- **AZD-2171:** Production of anti-angiogenic drug (WO20050004871, WO2005004872), composed of maleate (WO2005061488), modulating the activity of p53 kinase (WO2006014290, WO2006081034), in combination with gemcitabine (WO2007003933), Mek-inhibitor II (WO2008125820).
- **Bevacizumab (BV):** BV in combination with various treatments such as ZD6474 (WO2008037996), campthotecin (WO2010043050), carbonic hydrate (WO2013130354), pyradizanie derivatives (WO2013139423), a parvovirus (WO2016128146), AMP (WO2017045595), immune-conjugates that bind to FORLI (IMGN853) and doxorubicin (WO2017049149), ultrasounds (WO2017080481); BV administration method to increase BV penetration in the brain (WO2011049906); BV preparation with enhanced stability comprising buffering agent and osmotic pressure regulator (WO2016045570).
- **CBL-0137:** Method of production of CBL-0137 and use for cancer treatment (WO2015157172).
- **CDX-110:** Fabrication and use of antibody vaccine that preferentially binds to GPNMB, MET, EGFR, ALK (tyrosine kinase receptor), and induce immune antitumor activity (WO2004074432, WO2010135547, WO2016149265, WO2016168634), peptide-vaccine composition containing KL-H-peptide conjugate (WO2011077309).
- **DCVax-L:** Method to increase class I presentation of antigens by human dendritic cell (DC) (WO2001087325), methods to isolate, cultivate, differentiate DC precursor to form immature and/or mature DC preferentially to trigger T1 immune response (WO20030110292, WO2004072262, WO2004076651, WO20067067782, WO2017004230, WO2017048875, WO2003022215, WO2003095668), tangential flow filtration method to remove and isolate leukocyte from patient's blood (WO2004000444), composition comprising dendritic cells for administration to a patient (WO2004053072).
- **Enzastaurin:** Use of Enzastaurin in combination with HDAC inhibitor to treat cancer (WO2010074936).
- **GDC-0084:** phosphoinostide/pyrimidine kinase inhibitor and use for anticancer treatment (WO2007127183, WO2009042607).
- **Gliadel:** Carmustine alone or in combination with other drugs, with/without specific solvent, lyophilized or not, for cancer treatment (WO2003049743, WO200811960, WO2016077406), system for releasing carmustine in the brain using wafer/implant (WO2008013709, WO2016095592).
- **Gliovac:** Tumor vaccine comprising allogenic or xenogeneic tumor cells (WO2007085648).
- **H1-PV:** Method of tumor treatment using the parvovirus H1PV (US20120237483, WO2011157447, WO2012052158, WO2016206807, WO2016206844).
- **ICT-107:** Method of cancer treatment by dendritic cell vaccination comprising tumor associated antigens (US8097256, WO2014127296).
- **IMA950:** gp96 carrying antigens to activate DC (WO2002004516), or tumor associated peptides with/without tumor-associated T-helper cell peptide epitotes derived (or not) from survivin, preferentially binding to MHC-I (WO2003102023, WO2004085461, WO2005076009, WO2005116051, WO2009015841, WO2009015842, WO2009015843, WO2009138236, WO2010037513, WO2010037514, WO2015018805, WO2016102272, WO2016146751, WO2016156202, WO2016156230, WO2016170139, WO2016177784, WO2016202963, WO2016207164, WO2017001491, WO2017005733, WO2017009400, WO2017021527, WO2017060169, WO2017097602, WO2017097699, WO2017108345, WO2017140897, WO2017148888, WO2017157928, WO2017157972, WO2017174645, WO2017202806), generating immune antitumor activity.
- **MEDI-3617 and MEDI-575:** antibody association with sucrose to prevent antibody self-association (WO20122003470).
- **Mibefradil:** Method of preparation of Mibefradil (WO1998049147, WO1998049148, WO1998049149), anti-metastatic activity of Mibefradil (WO2005086971).
- **Nanocell/targoMir:** Method for purifying bacterial minicells (WO2004113507), method for targeting mammalian cells with minicells (WO2005079854, WO2006021894, WO2009027830), minicells brain tumor targeting (WO2013088250), combined treatment with minicells and interferon-gamma (WO2015049589).
- **NOX-A12:** Spiegelmer, in some cases immobilized (WO2001092566, WO2003035665).
- **Prophage:** Composition comprising a heat shock protein and a saponin or an antigen (WO2002011669, WO2004091493).
- **SapC-DOPS:** Composition comprising combination of saposin C and dioleoylphosphatidylserine for tumor treatment (WO2004096159).
- **SurVaxM:** surviving peptide vaccine for tumor treatment (WO2000003693, WO2006081826, WO2007036638, WO2007039192, WO2009012460, WO2009138236, WO2014153636, WO2016179573).
- **TC-A237:** Combination of Mek and Aurora inhibitors (WO2012167247).
- **TMZ:** Combined antitumor treatment with TMZ and ATase inhibiting agent (WO1994015615), Cisplatin (W01997007804), interferon (WO1997012630, WO2001052882), immunocytokine (WO20100078916), VEGFR2 (WO2010093771), methoxyamine (WO2001012199), irinotecan (WO2001054678), thalidomide (WO2002043720), TNF-ALPHA (WO2006026348), kinase inhibitors (WO2007033374, WO2008094484), bormeol and/or methol (WO2008022535), TMZ administered in microcrystalline suspension (WO2000033823), cancer treatment method with TMZ (WO2000057867), methods for TMZ synthesis (WO2002057268, WO2002057269), controlled release system containing TMZ (WO2004028534), various formulations/compositions of TMZ and TMZ derivatives (WO2005063757, WO2006024238, WO2006032190, WO2008111092, WO201040168, WO2011036676, WO2012013116, WO2014091078, WO2014104671, WO2015062481), various method of TMZ protocol/administration/dosage for cancer treatment (WO2006060464, WO20080002544, WO2008038031, WO2008140724, WO2011072240, WO2011077458).
- **TOCA-511:** Formulation containing 5-fluorocytosine and/or retroviral vectors with immune-stimulating activity for cancer treatment (WO2010002937, WO2010148203, WO2011126864, WO2012058637, WO2014201449, WO2015021077, WO2015148683, WO2017040815).
- **Val-083:** Various derivatives of hexitols for cancer treatment alone or in combination with other drugs (WO2001091741, WO2012024367, WO2012024368, WO2013110058, WO2013128285, WO2014004376, WO2014194312, WO2016077264, WO2016183331, WO2017042634, WO2017075052, WO2017091588).

- **VB-111:** Fas-chimera adenovirus vector for cancer treatment (US9200056).

## The different actors tackling GBM disease

**Figure 4:**
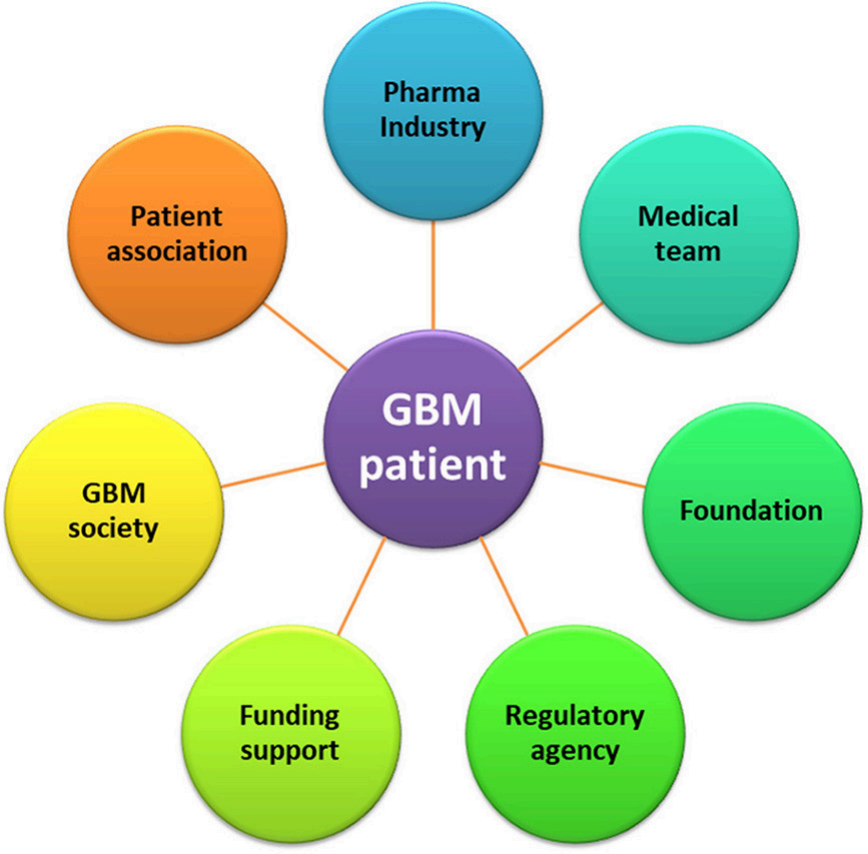
A schematic diagram presenting the GBM community fighting against GBM, at the heart of which lie the patients.

At the heart of the GBM community lie the patients. Different structures contribute to the effort for the development of an effective GBM treatment. They consist of medical teams and hospital services dedicated to GBM treatment, EANO and SNO associations that organize conferences on glioblastoma and various means of communication within the glioblastoma community, patient associations, foundations, pharmaceutical companies, regulatory agencies, and various public and private structures that provide funding for research and clinical trials (Figure 4).

## GBM treatment cost

GBM costs can be divided between direct costs due to stays and treatments carried out at hospital and indirect costs coming for example from work leave and resulting income losses. In the United-States, GBM average direct cost per patient has been estimated as 8,500 $ per month, mainly coming from surgery, imaging, and radiotherapy, while standard chemotherapy only represents 0.1% of this cost (Cagney and Alexander, 2017). Direct costs have been shown to strongly depend both on country, varying from an average of 27,000 $ per patient in Sweden to 95,000 $ per patient in the United-States (Raizer et al., 2015), and on the type of given care, for example being less expensive using brachytherapy (23,000 $/patient) than external beam therapy (33,000 $/patient) (Raizer et al., 2015). Importantly, indirect GBM costs are usually reported to be much higher than direct ones, being 101,000 $/patient in Sweden (Raizer et al., 2015) and 112,000 $/patient in Spain (Undabeitia et al., 2018). Another important issue relates to treatment benefit relative to its cost. This can be evaluated by measuring the so-called incremental cost-effectiveness ratio per life of year gained (LYG). For cancer an acceptable average threshold has been set at 50,000 $/LYG (Raizer et al., 2015). For glioblastoma, which are extremely difficult to treat, this threshold is often exceeded, yielding 70,000 $/LYG for TMZ, 115,000 $/LYG for carmustine wafer, and 550,000 $/LYG for TTF (Raizer et al., 2015; Cagney and Alexander, 2017). Whereas, such high costs may be justified for TMZ and TTF, since both of these treatments increase PFS by 2 and 4 months, respectively (Stupp et al., 2005; Cagney and Alexander, 2017), it does not seem to be the case for carmustine wafers that have not demonstrated survival benefit and produce severe side effects (Bregy et al., 2013). Bevacizumab was also reported to lack cost effectiveness for treating GBM patients (Kovic and Xie, 2015).

## Market

GBM market was estimated as 465 million $ in 2016 and is expected to reach 1 billion $ by 2025, being equally distributed between the United States, Europe, and Asia and the rest of the world (Glioblastoma Multiform market 2024, GBM Industry Research Report, Hexa research, California, United-States).

## Analysis of companies developing GBM treatments

Table 3 summarizes financial information concerning the various companies developing GBM therapies. Financial analysis has been carried out on companies that devote a substantial part of their activity to developing a GBM treatment, i.e., companies that mention GBM as therapeutic target in their 2017 annual report. Sixty percent of these companies fall within the category of small businesses (<100 employees), a quarter of them are of medium sizes (between 100 and 500 employees), and 15% of them employ more than 500 people. Only the large companies seem to generate revenues. For Elekta, this may be due to the development of a medical device (Gamma-Knife) with less stringent regulations than a drug, multiple possible uses on various indications, and a marketing approach relying on selling a therapeutic device only once to a hospital, hence significantly reducing costs of fabrication and selling prices compared with drugs. On the other hand, Roche sells Avastin, a drug against GBM that has already been accepted for commercialization and can therefore be sold without substantial additional investment. Despite of the financial success of these large companies, the treatments that they commercialize do not enable to treat efficiently GBM. More efforts in research and development (R&D) should therefore be spent to improve this situation. Today most R&D financial investment on new GBM treatments is carried out by small and medium size companies with a distinction to be made between those concentrating exclusively on GBM (Immuno Cellular, Tocagen, Northwest Biotherapeutics, Del Mar Pharmaceuticals) and those with a more diverse portfolio of targeted indications (Agenus, Celldex, CytRx, Midatech, Novocure). These companies have been founded between 11 and 26 years ago, a lapse of time that has enabled most of them to reach clinical trials but was insufficient to yield business profitability. Indeed, all these companies incur losses, between 41 and 905 m$, and have a market capitalization that is lower than their accumulated losses. Furthermore, only Novocure seems to generate substantial revenues with its GBM treatment. This may be due to the relative efficacy of its Optune treatment observed in a phase III clinical trial carried out on GBM patients. Interestingly, our analysis does not lead to the conclusion that companies with a more diverse portfolio of targeted indications have a better financial situation than those mainly focusing on GBM. In fact, treatments against other cancers than glioblastoma may be less difficult to develop, but still require a significant amount of time and investment to reach commercialization, which opponently have not yet been reached by these companies. Our analysis further seems to suggest that development time, total financial investment, level of complexity, and benefit/risk ratio of the drug/medical device under development, are the parameters that determine if/when a company developing a GBM treatment can reach profitability.

**Table 3:** Financial information concerning the various companies developing or commercializing GBM treatments, extracted from the 2017 annual report of these companies.

**Co name**	**Year founded**	**Location (HQ) 2016**	**Revenue (MS, 2016)**	**Net income/Loss (MS, 2016)**	**Accumulated losses (MS)**	**No. of employees 2016**	**R & D (% GBM) (MS, 2016)**	**Gal & Admin (MS, 2016)**	**Market value (MS, 2018)**
Agenus	1994	Lexington (USA)	22	−127	905	255	94 (8% GBM)	33	516
Celldex	2005	Hampton (USA)	7	−128	719	210	103 (10% GBM)	36	317
CytRx	1985	San Francisco (USA)	0.2	−51	416	27	36 (<10% GBM)	16	243
Deciphera Pharma	2003	Waltham (USA)	0	−12	176	42	14 (<15% GBM)	4	57
DelMar Pharma	2009	Vancouver (Canada)	0	−9	41	4	5 (100% GBM)	3	23
Elekta	1972	Stockholm (Sweden)	11	0.1	N.A.	3,600	0.15 (>50% GBM)	0.09	N.A.
Immuno-cellular	1987	Los Angeles (USA)	0	−27	96	7	19 (100% GBM)	5	4.2
Midatech	2000	Oxford (UK)	6.4	−20	59	84	6.7 (15–30%)	9	22
Northwest biotherapeutics	1996	Bethesda (USA)	0.6	−80	715	15	60 (100% GBM)	11	N.A.
Novocure	2000	Jersey Isle	83	−131	520	450	41 (20% GBM)	51	N.A.
Noxxon	1997	Berlin (Germany)	0.083	−11	129	10	5 (<20% GBM)	4	12.3
Roche	1896	Basel (Switzerland)	54,000	8,825	NA	94,000	10,400 (<5% GBM)	NA	190,988
Tocagen	2007	San Diego (USA)	0.031	−28	156	61	21 (100% GBM)	6	215

*The year of creation of these companies, the location of their headquarter, their revenues in 2016, their net incomes or losses in 2016, their accumulated losses since their foundation, their number of employees in 2016, their amount of spending in R&D in 2016 with the estimated percentage of this spending dedicated to GBM research, their administrative and general spending in 2016, as well as the market value of these companies in 2016, are indicated. The market value was estimated by multiplying the value of the share by the number of shares for each of these companies. The percentage of R&D spending dedicated to GBM research was estimated by dividing the number of GBM drugs by the total number of drugs developed/commercialized by each of these companies. This estimate relies on the analysis of the 2017 annual report of these companies, possibly not mentioning some drug developments, hence possibly leading to overestimate/underestimate of this percentage*.

## Conclusion and future perspective:

Glioblastoma is a very aggressive cancer, leading to patient death a few months only following diagnosis. For operable GBM, surgery remains the most effective initial GBM treatment. However, it does not enable the removal of the entire tumor and the tumor therefore re-grows.

GBM treatments that are under development or commercialized include:

- Methods to improve surgery, such as the maintenance of GBM patients awake during the surgical operation (AWC), the use of a robotized system enabling to improve surgery accuracy (Neuroarm), tools to improve visualization of tumor cells and enable a distinction between tumor and healthy cells using fluorescence imaging (PET, OCT, CLEM), magnetic imaging (iMRI, gMRI, MEG, nTMS), DTI-FT or MS.
- Techniques to improve radiotherapy, using an external X-ray source, which is combined with tumor imaging (IGRT, HT), modulation of radiation intensity (IMRT), a focalization of the radiation beam at some specific locations of the tumor (SRT, Gamma-knife, cyber-knife), an external source of protons enabling to limit the overlap of the radiation beam with the healthy tissue region (PRT), an internal source of X-rays (BT, RmAB, RS).
- GBM treatments using different electromagnetic radiation sources such as the electric field blocking the mitosis of tumor cells (Optune), or laser thermotherapy locally heating the tumor (Neuroblate, Visualase).
- Therapies targeting specific parts of the tumor (Mibrefadil, TMZ, Gliadel, Aldoxorubicin, Val-083, ANG-1005, Afatinib, CBL0137).
- Drugs against angiogenesis (Bevacizumab, Altiratinib, MLN518, SapC-DOPS, VB-111, Enzastaurin, TC-A237, AZD2171).
- A kinase inhibitor (GDC-0084)
- Immunotherapies such as vaccines (Rindopepimut, SurVaxM, Prophage, Gliovac, IMA950, DCVax-L), antibodies (Depatux-M, Asunercept, MEDI-3617 and MEDI-575), check point inhibitors (NOX-A12).
- Nanotherapies (Nanocell, AuNP)
- miRNA targeting (TargoMIR)
- Glioma stem cell targeting (ICT-107)
- Gene Therapy (TOCA511)
- Virus (ParvOryx)

Among these treatments, Optune seems to be the only one with some efficacy (although rather modest) demonstrated in a phase III clinical trial. Many of them are still at a too early stage of development to be able to firmly conclude about their efficacy.

Several ways to improve the efficacy of GBM treatments have also been suggested. Early diagnosis methods could be developed enabling the treatment of smaller tumors possibly easier to eradicate. More preclinical trials could be carried out on large animals such as dogs whose relatively large tumor sizes could lead to a better estimate of the human dose than mouse studies. Drug delivery could be improved to better enable GBM drugs to reach the tumor. For intravenous injection, new methods shall be developed to allow GBM drugs to cross the BBB. For intra-tumor administration, a better diffusion of the drug should be obtained, for example by using CED.

At an industrial level, the development of GBM therapies has been facilitated by the orphan drug status that applies on GBM drugs due to the low prevalence of GBM. Several analyzed companies seem to earn a large number of patents protecting their GBM treatment and may be able to generate revenues when/if they firmly demonstrate some efficacy with their GBM drug, as it is the case for Novocure that has announced a large revenue in 2016 (Table 3).

## Expert opinion section

This review describes industrial developments of GBM drugs and medical devices at different stages of developments, i.e., which were tested in:

- early clinical trials not yet enabling to conclude about their efficacy on a large cohort of patients (ICT-107, VAL-083, Depatux-M, MgLITT, Prophage, APG101, Mibefradil, Nanocell, ERC-1671, IMA950, MEDI-575, Panobinostat, Survax-M, DC-Vax-M, Parvovirus, Gama knife).
- phases II or III clinical trials resulting in an absence of efficacy (Rindopepimut, Avastin, Gliadel, PSMA ADC, Trebanaib, Afatinib, Enzastaurin, Tandutinib).
- phase III clinical trials demonstrating some modest efficacy (Temozolomoide, Optune)
- pre-clinically mainly showing tumor growth retardation on tumors originating from PDX and/or immortalized cell lines (BiCNU, AV-0113, GMCI, AFM21, ANG1005, SapC-DOPS, Aldoxorubicin, Altiratinib, CBL0137, Selenexor, Indoximod, GDC-0084, NOX-A12, Parvovirus, Toca 511, VB-111).
- Cells showing decrease in GBM cell survival or proliferation (Crenolanib, KML001, TC-A2317)

Optune is the only recently developed GBM drug that has shown some efficacy (although rather modest) on a large cohort of patients.

Among the approaches tested for GBM treatments that have led to preclinical efficacy or clinical efficacy on a limited number of patients are:

- drugs targeting of various types of molecules such as T-type channel (Mibefradil), DNA (Aldoxorubicin, Val-083, CBL0137), microtubule (ANG-1005), EGFR (Afatinib),
- Anti-angiogenic drugs (Altiratinib, SapC-DOPS, VB-111, Alisertib,
- Kinase inhibitor (GDC-0084),
- Immunotherapies (Survax-M, Prophage, Gliovac, IMA950, DCVax-L, Asunercept, NOX-A12
- Nanotherapies (Gold nanoparticles)
- Glioma stem cell targeting (ICT-107)
- Gene therapy (TOCA 511 combined with TOCAFC)
- Virus (ParvOryx)

More clinical trials are necessary to determine if these drugs are efficient (or not) on a large number of patients.

Traditional approaches fail to treat efficiently glioblastoma. Surgery does not completely remove glioblastoma without damaging the brain. Radiation therapy cannot be used beyond a certain threshold dose, which is insufficient to completely eradicate glioblastoma. Chemotherapy has shown limited efficacy and can be very toxic.

According to the author, one of the most interesting therapeutic approaches is to expose the tumor to an external energy source, such as an alternating electric or magnetic field, to repeatedly induce antitumor activity. Ideally, these sources should be chosen to be able to carry out the treatment until the tumor has fully disappeared. They should also be sufficiently compact, inexpensive, and easy to use so that patients can carry out the treatment at home, possibly with the help of a nurse. Ultimately, it is desirable that the use of the hospital environment is minimized to reduce costs and allow the treatment of as many patients as possible at a reasonable cost for each patient.

Immunotherapy approaches have also raised an enormous interest, but failed until now to show antitumor efficacy on humans. This may be due to the complex immune mechanisms that are not yet fully described and understood. These approaches should be pursued, maybe by trying to reactivate the immune system against the tumor several times until the tumor has fully disappeared.

Finally, on the one hand more effort should be spent to develop a proper preclinical model, without which treatment efficacy cannot be properly assessed. On the other hand, methods should be developed to diagnose glioblastoma earlier. The author thinks that those steps are prerequisites to develop an efficient glioblastoma treatment.

## Author's note

Due to the proximity of the author with the two companies Nanobacterie and Magforce, the latter are not analyzed in this review. The patents were quoted by their publication number.

## Author contributions

The author confirms being the sole contributor of this work and approved it for publication.

### Conflict of interest statement

EA has been working with the company Nanobacterie.

## Glossary (non-exhaustive)

Allogeneic antigen, Antigen occurring in some but not all patients;

Anaplastic astrocytoma, tumors developing from brain cells called astrocytes;

Anaplastic ependymoas, tumor that forms when cells in the central nervous system (including the brain and spinal cord) begin to multiple rapidly;

Autologous antigen, Antigen belonging to the same organism;

Brain parenchyma, brain nervous tissue;

Cerebral edema, excess accumulation of fluid in the brain;

Cerebrospinal fluid, regulates the distribution of substances between cells of the brain;

Cortical, belongs to the cerebral cortex is the largest region of the cerebrum in the mammalian brain and plays a key role in memory, attention, perception, cognition, awareness, thought, language, and consciousness;

Dendritic cells (DC), Antigen-presenting cells whose function is to process antigen material and present it on the surface to T cells of the immune system;

Glial cells, cells consisting of microglia, astrocytes, and oligodendrocyte lineage cells as their major components, constitute a large fraction of the mammalian brain;

Hippocampi, parts of the brain responsible for memory;

Microtubules, Parts of the cytoskeleton of cells;

Oligdendroglioma, third most common type of glioma, comprising 4%–15% of all glioma, and classified by their degree of malignancy into grades II or III, according to WHO classification. Only 30% of oligodendroglial tumors have anaplastic characteristics;

Overall survival (OS), Percentage of patients who are still alive for a certain period of time after they were diagnosed with a disease or started treatment for a disease, such as GBM;

Platelet-derived growth factor receptors (PDGFR), Cell surface tyrosine kinase receptors of platelet-derived growth factors (PDGF), which regulate proliferation, differentiation, and growth of cells;

Progression free survival (PFS), Length of time following a treatment during which a disease does not worsen;

Ventricle, communicating network of cavities filled with cerebrospinal fluid (CSF) and located within the brain parenchyma.
